# GlcNAc induces GlcNAc catabolic genes and inhibits filamentation via YlRep1-YlNgs1 signaling in the dimorphic yeast *Yarrowia lipolytica*

**DOI:** 10.1128/msphere.00477-25

**Published:** 2025-10-03

**Authors:** Zhen-Hua Wang, Meng-Yang Xu, Xiang-Dong Gao

**Affiliations:** 1 Hubei Key Laboratory of Cell Homeostasis, College of Life Sciences, Wuhan University, Wuhan, China; 2 Division of Natural and Applied Sciences, Duke Kunshan University 517759, Kunshan, China; University of Guelph, Guelph, Ontario, Canada

**Keywords:** GlcNAc catabolism, filamentous growth, filamentation, dimorphic transition, dimorphism

## Abstract

Many fungal species can utilize *N*-acetylglucosamine (GlcNAc) as a carbon source. Studies in the pathogenic yeast *Candida albicans* have revealed that GlcNAc utilization and the induction of GlcNAc catabolic genes depend on the Ndt80 family transcription factor CaRep1 and the histone acetyltransferase CaNgs1. Additionally, GlcNAc induces filamentation via both alkalinization of the medium and CaNgs1 signaling. However, the roles of YlRep1 and YlNgs1 in GlcNAc catabolism and filamentous growth are not clear in the dimorphic yeast *Yarrowia lipolytica*. In this study, we demonstrate that YlRep1 and YlNgs1 are essential for the induction of GlcNAc catabolic genes in *Y. lipolytica*, which is similar to the function of CaRep1 and CaNgs1. YlRep1 and YlNgs1 interact physically and exhibit transcriptional activation activity on a reporter gene. Interestingly, unlike in *C. albicans*, GlcNAc inhibits filamentation in *Y. lipolytica*. This inhibition requires YlRep1-YlNgs1 but does not depend on the alteration of ambient pH. We show that YlRep1 and YlNgs1 co-repress a set of transcription factor and cell wall protein genes, some of which are associated with filamentation. Notably, this repression is independent of GlcNAc catabolism but requires the GlcNAc kinase, YlNag5.

**IMPORTANCE:**

GlcNAc has been used previously to induce filamentation in *Yarrowia lipolytica*, but often in combination with a citrate buffer at near-neutral pH. The exact role of GlcNAc in regulating filamentous growth is unclear. In this study, we report that GlcNAc inhibits rather than promotes filamentation in *Y. lipolytica*, and this function does not require GlcNAc catabolism or the alteration of ambient pH by GlcNAc catabolism. We show that YlRep1-YlNgs1 signaling, which activates GlcNAc catabolic genes, represses a set of filamentation-related genes and is a key regulator in the inhibition of filamentation by GlcNAc. This finding indicates that YlRep1-YlNgs1 has dual roles, functioning both in the activation of GlcNAc catabolic genes and the repression of filamentation-related genes in response to GlcNAc. These findings provide new insights into the regulatory mechanisms of GlcNAc catabolism and signaling in *Y. lipolytica*.

## INTRODUCTION

*Yarrowia lipolytica* is a nonconventional yeast with genomic, physiological, and metabolic characteristics that differ from those of the model yeasts *Saccharomyces cerevisiae* and *Candida albicans* (1). It has a wide range of industrial applications in the production of heterologous proteins, organic acids, terpenes, and biofuels (2, 3). Like other dimorphic yeasts, *Y. lipolytica* can switch from oval-shaped yeast form to filamentous forms, which is induced by environmental factors, such as carbon source, nitrogen source, temperature, pH, low oxygen, or osmotic pressure (4, 5). Dimorphic transition is considered a strategy for fungi to acquire nutrients and adapt to the environment (6). For some pathogenic fungi, such as the human pathogen *C. albicans*, the formation of hyphae promotes invasion into tissues and biofilm formation (7, 8).

*Y. lipolytica* can utilize different carbon sources, and cells exhibit different morphologies depending on the type of carbon sources available in the environment. In the presence of carbon sources, such as oleate, olive oil, and castor oil, *Y. lipolytica* cells typically remain in the oval-shaped yeast form (9, 10). However, when exposed to carbon sources, such as glucose, fructose, peptone, lactate, and citrate, *Y. lipolytica* cells elongate and develop filaments (5, 10). The molecular mechanisms that govern this morphological transition remain unclear. A recent study suggests that glucose-induced filamentation in *Y. lipolytica* is regulated by sugar signaling pathways, most likely via the cAMP-PKA-dependent pathway (11). Among the common carbon sources, glycerol is a preferred choice for *Y. lipolytica*, and cells grown in glycerol are in the yeast form (10). The conserved nutrient-sensing TORC1-Sch9 signaling pathway is involved in the inhibition of filamentation by glycerol (10).

*N*-acetylglucosamine (GlcNAc) is another carbon source that *Y. lipolytica* can utilize. This amino sugar is the building block of important biological molecules, such as cell wall peptidoglycan in bacteria, cell wall chitin in fungi and parasites, exoskeletons of arthropods, and the extracellular matrix of animal cells (12, 13). GlcNAc can be utilized as a carbon source and nitrogen source by most bacteria and fungi, except for some species such as the model yeasts *S. cerevisiae* and *Schizosaccharomyces pombe,* because they lack GlcNAc catabolic genes (13). In fungi, GlcNAc is catabolized to fructose-6-phosphate in a stepwise manner by GlcNAc transporter, GlcNAc kinase, GlcNAc-6-phosphate deacetylase, and glucosamine-6-phosphate deaminase, which are encoded by *NGT1*, *HXK1*/*NAG5*, *DAC1/NAG2*, and *NAG1*, respectively (13–15). The phosphorylated form of GlcNAc, GlcNAc-6-phosphate, is a precursor of uridine diphosphate-GlcNAc (UDP-GlcNAc), which is important for synthesizing cell wall chitin in fungi and the extracellular matrix in animal cells, as well as other cellular processes. There is a reverse pathway to synthesize GlcNAc-6-phosphate from fructose-6-phosphate, which is important when GlcNAc is absent in the environment (13).

In addition to the roles of an energy source and components of cell-surface structure, extracellular GlcNAc is also a signaling molecule. GlcNAc can downregulate the production of curli, extracellular surface fibers that function in biofilm formation in the bacteria *Escherichia coli* (16). In animal cells, increased GlcNAc can stimulate O-linked attachment of GlcNAc to nuclear and cytoplasmic proteins, which modulates signaling and influences protein expression, degradation, and trafficking (17). In the human pathogen *C. albicans*, GlcNAc induces filamentation, white-opaque transition, and GlcNAc-induced cell death (15). GlcNAc is also a potent inducer of the yeast-to-filament transition in two thermally dimorphic fungi, *Histoplasma capsulatum* and *Blastomyces dermatitidis* (18).

The fungal GlcNAc sensor Ngs1 was first discovered in *C. albicans* (19). It is an *N*-acetyltransferase related to the *S. cerevisiae* histone acetyltransferase Gcn5. GlcNAc binds to CaNgs1 and activates its *N*-acetyltransferase activity. CaRep1, a negative regulator of *MDR1* transcription, is essential for growth on GlcNAc in *C. albicans* (19, 20). It is an Ndt80 family transcription factor, which also includes CaNdt80 and CaRon1 (21, 22). Because CaNgs1 does not bind to DNA, CaRep1 recruits CaNgs1 to the promoters of target genes, where CaNgs1 activates gene expression by histone acetylation (19). The Rep1-Ngs1 signaling is essential for GlcNAc utilization and the induction of GlcNAc catabolic genes in response to GlcNAc. In the filamentous fungus *Trichoderma reesei*, Ngs1 and the transcription factor Ron1 (ortholog of *C. albicans* CaRep1 but not CaRon1) are also essential for GlcNAc catabolism (23, 24).

In addition to GlcNAc utilization, CaNgs1 but not CaRep1 is also important for GlcNAc-induced filamentation (19, 22). Ca*ngs1*Δ cells and Ca*ngs1*Δ Ca*hxk1*Δ cells were severely defective in hyphal development in medium containing both GlcNAc and galactose. Moreover, CaNgs1 is essential for the induction of hyphal-specific genes by GlcNAc (19). In *Candida tropicalis*, which is closely related to *C. albicans*, GlcNAc strongly inhibits hyphal growth (25), and this inhibition requires CtRep1 (26). The Ndt80 family transcription factor CtRon1 also regulates filamentation. However, it is required for serum-induced filamentation but not for the inhibition of filamentation by GlcNAc (26).

Here, we show that, similar to *C. albicans*, YlRep1-YlNgs1 is necessary for the induction of GlcNAc catabolic genes in response to GlcNAc in *Y. lipolytica*. However, unlike in *C. albicans*, GlcNAc inhibits filamentation in *Y. lipolytica*. This inhibition is independent of GlcNAc catabolism but requires both YlRep1-YlNgs1 and the GlcNAc kinase YlNag5. Therefore, YlRep1-YlNgs1 plays dual and opposing roles in GlcNAc-regulated catabolism and dimorphic transition in *Y. lipolytica*.

## RESULTS

### *Y. lipolytica* shares a similar GlcNAc catabolic pathway and YlRep1-YlNgs1 regulation of GlcNAc catabolic genes with *C. albicans*

**Fig 1:**
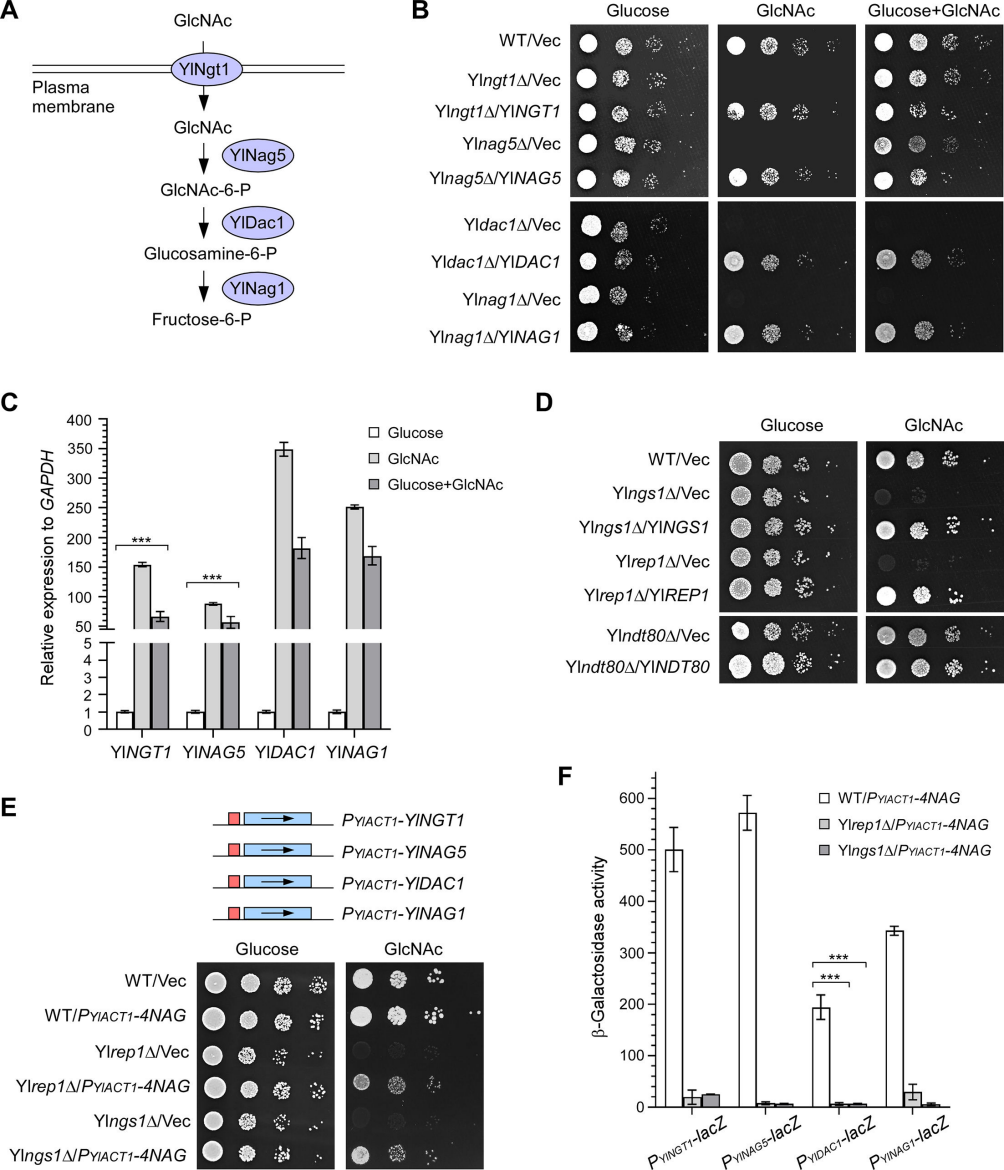
GlcNAc induces GlcNAc catabolic genes in a YlRep1- and YlNgs1-dependent manner in *Y. lipolytica*. (**A**) The GlcNAc catabolic pathway in *Y. lipolytica*. (**B**) Yl*NGT1*, Yl*NAG5*, Yl*DAC1*, and Yl*NAG1* genes are essential for growth in GlcNAc medium. Wild-type (WT), Yl*ngt1*Δ, Yl*nag5*Δ, Yl*dac1*Δ, and Yl*nag1*Δ cells carrying pINA445 (Vec) or pINA445-Gene were spotted at 1:10 serial dilution on YNB agar containing the indicated sugar. Pictures were taken after 2 days at 30°C. (**C**) GlcNAc induces the expression of Yl*NGT1*, Yl*NAG5*, Yl*DAC1*, and Yl*NAG1* genes. The transcription levels of these genes in wild-type cells grown in liquid YNB medium containing the indicated sugar were determined by qRT-PCR and normalized to glyceraldehyde-3-phosphate dehydrogenase (*GAPDH)*. (**D**) GlcNAc catabolism depends on YlRep1 and YlNgs1 but not YlNdt80. Wild-type (WT), Yl*ngs1*Δ, Yl*rep1*Δ, and Yl*ndt80*Δ cells carrying pINA445 (Vec) or pINA445-Gene were spotted at 1:10 serial dilution and grown on YNB-Glucose and YNB-GlcNAc agar for 2 d at 30°C. (**E**) Yl*ngs1*Δ and Yl*rep1*Δ cells carrying *P_YlACT1_-4NAG* grow in GlcNAc medium. Wild-type (WT), Yl*rep1*Δ, and Yl*ngs1*Δ cells carrying pYL27 (Vec) or pYL27-4NAG (*P_YlACT1_-4NAG*) were spotted at 1:10 serial dilution and grown on YNB-Glucose and YNB-GlcNAc agar for 2 days at 30°C. The plasmid pYL27-4NAG carries Yl*NGT1*, Yl*NAG5*, Yl*DAC1*, and Yl*NAG1* genes under the control of the Yl*ACT1* promoter. (**F**) YlRep1 and YlNgs1 are essential for the induction of GlcNAc catabolic genes by GlcNAc. Wild-type (WT), Yl*rep1*Δ, and Yl*ngs1*Δ cells carrying both pYL25-4NAG (*P_YlACT1_-4NAG*) and pINA445-P_Gene_-*lacZ* were grown in YNB-GlcNAc medium. β-Galactosidase activities of *promoter-lacZ* constructs were measured. All the spot plate assays were done at least three times on different days. For qRT-PCR and β-galactosidase assays, mean data ± standard deviation from three independent experiments carried out on different days were plotted. The unpaired two-tailed Student’s *t*-test was used to examine the statistical significance of the difference between two samples. Statistically significant differences are indicated by the asterisks (***, *P* < 0.001).

Similar to *C. albicans*, GlcNAc catabolism in *Y. lipolytica* is thought to require stepwise reactions catalyzed by YlNgt1, YlNag5, YlDac1, and YlNag1 (14) (Fig. 1A). We found that Yl*ngt1*Δ, Yl*nag5*Δ, Yl*dac1*Δ, and Yl*nag1*Δ cells failed to grow in GlcNAc medium but grew well in glucose medium (Fig. 1B), indicating that the four proteins are essential for GlcNAc utilization. Yl*dac1*Δ and Yl*nag1*Δ cells also failed to grow in the medium containing both glucose and GlcNAc (Fig. 1B), resembling the growth defect observed in *C. albicans* Ca*dac1*Δ and Ca*nag1*Δ cells (27). This finding suggests that the accumulation of metabolic intermediates is toxic to the cells. Similar to what is observed in *C. albicans*, GlcNAc strongly induced the expression of Yl*NGT1*, Yl*NAG5*, Yl*DAC1*, and Yl*NAG1* genes in *Y. lipolytica* (Fig. 1C). Interestingly, the strong induction of these genes by GlcNAc persisted even in the presence of a high concentration of glucose (1%). These results suggest that *Y. lipolytica* shares a similar GlcNAc catabolic pathway with *C. albicans*.

In *C. albicans*, the histone acetyltransferase CaNgs1 serves as the GlcNAc sensor and transducer (19). CaNgs1 is constitutively targeted to the promoters of GlcNAc-inducible genes via the Ndt80 family transcription factor CaRep1, and both CaNgs1 and CaRep1 are required for the transcriptional activation of GlcNAc catabolic genes (19, 21). We identified YlNgs1 (YALI0E20185p, 28.0% identity) and YlRep1 (YALI0D24860p, 24.9% identity) as the *Y. lipolytica* orthologs of *C. albicans* CaNgs1 and CaRep1, respectively. *Y. lipolytica* also contains another Ndt80 family transcription factor, YlNdt80 (YALI0B14773p), which shares 34.0% and 31.8% amino acid sequence identity to CaNdt80 and CaRon1, respectively, but very low homology to CaRep1.

Similar to *C. albicans* Ca*rep1*Δ and Ca*ngs1*Δ cells, *Y. lipolytica* Yl*rep1*Δ and Yl*ngs1*Δ cells were severely impaired in growth on GlcNAc medium (Fig. 1D), indicating that YlRep1 and YlNgs1 may have similar roles in regulating GlcNAc catabolism as their orthologs in *C. albicans*. In contrast, Yl*ndt80*Δ grew well on GlcNAc medium (Fig. 1D), suggesting that YlNdt80 is not involved in GlcNAc catabolism.

To examine whether YlRep1 and YlNgs1 are required for the induction of GlcNAc catabolic genes by GlcNAc, and more importantly, to examine the morphology of Yl*rep1*Δ and Yl*ngs1*Δ cells grown in GlcNAc medium (see later section), we constructed a plasmid that carries Yl*NGT1*, Yl*NAG5*, Yl*DAC1*, and Yl*NAG1* genes, each of them was under the control of the strong constitutive promoter of Yl*ACT1* (encodes actin) (Fig. 1E, upper panel). As expected, growth was restored in Yl*rep1*Δ and Yl*ngs1*Δ cells carrying *P_YlACT1_-4NAG* in GlcNAc medium, although it was significantly slower than that of wild-type cells (Fig. 1E, lower panel). By using *promoter-lacZ* reporters, we found that *P_YlNGT1_-lacZ*, *P_YlNAG5_-lacZ*, *P_YlDAC1_-lacZ*, and *P_YlNAG1_-lacZ* reporters exhibited high levels of expression in wild-type cells grown in GlcNAc medium. In contrast, their expression levels were very low in Yl*rep1*Δ or Yl*ngs1*Δ cells carrying *P_YlACT1_-4NAG* (Fig. 1F). This result indicates that YlRep1 and YlNgs1 are essential for the induction of GlcNAc catabolic genes in response to GlcNAc.

Together, our results suggest that the GlcNAc catabolic pathway and YlRep1-YlNgs1 regulation of GlcNAc catabolic genes in *Y. lipolytica* are similar to those of *C. albicans*.

### YlRep1 and YlNgs1 interact physically, and YlRep1-YlNgs1 activates a reporter gene interdependently

**Fig 2:**
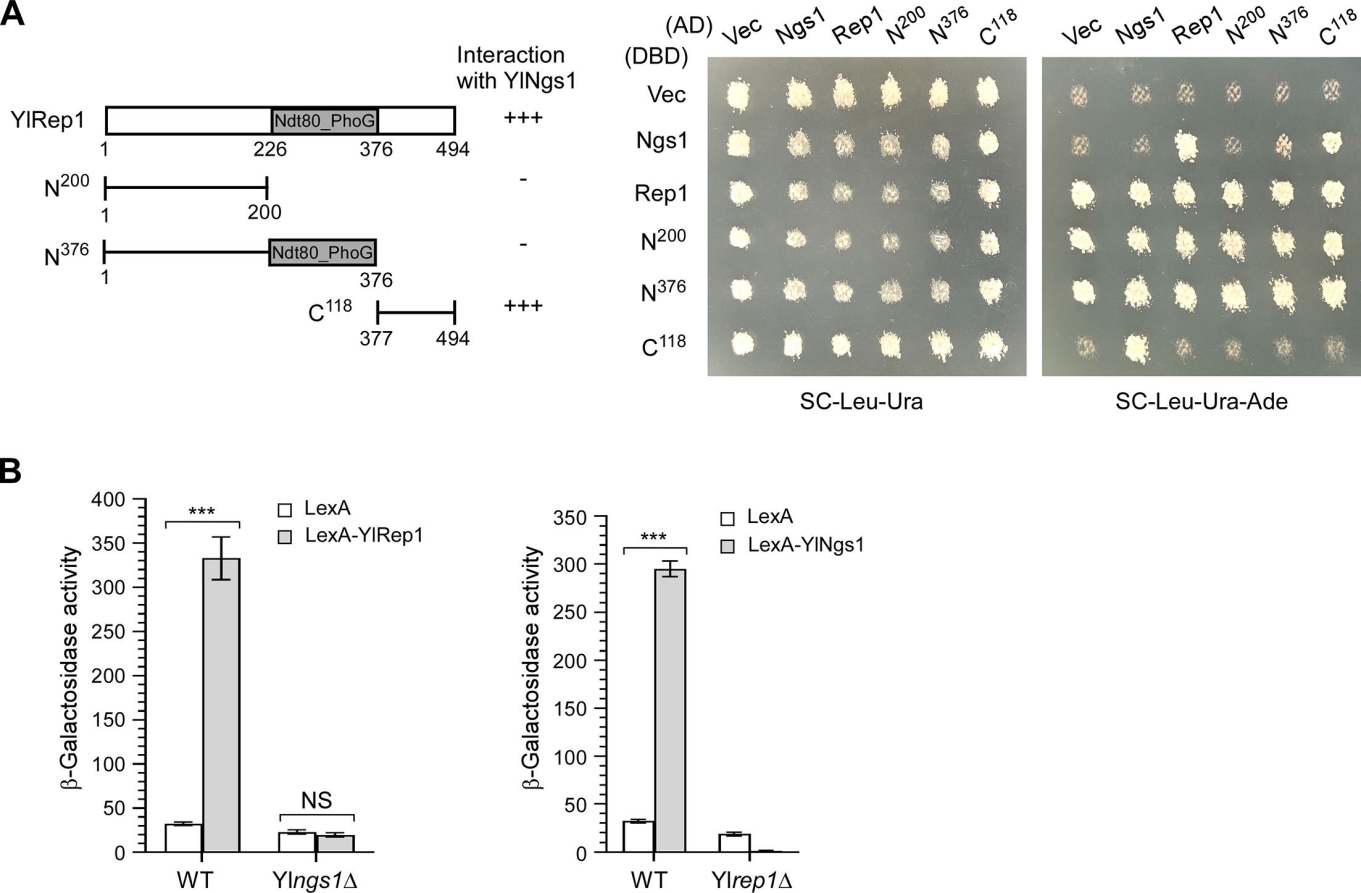
YlRep1 interacts with YlNgs1, and YlRep1-YlNgs1 activates a reporter gene in an interdependent manner. (**A**) Two-hybrid assay between YlRep1 and YlNgs1. pGBDU-C1 (Vector, containing DNA-binding domain, DBD), pGBDU-YlNGS1, pGBDU-YlREP1, and YlREP1 segments were paired with pGAD-C1 (Vector, containing activation domain, AD), pGAD-YlNGS1, pGAD-YlREP1, and YlREP1 segments. Cells were grown on an SC-Leu-Ura plate and then replica plated to an SC-Leu-Ura-Ade plate. Pictures were taken after 2 days at 30°C. The YlRep1 segments are depicted. The strength of interaction is indicated from strong (+++) to no effect (−). Note that full-length YlRep1, YlRep1-N^200^, and YlRep1-N^376^ exhibited auto-activation activity when fused to Gal4-DBD. The two-hybrid assay was done three times on different days. (**B**) YlRep1 and YlNgs1 activate *lexAop-P_YlLEU2_-lacZ* expression in an interdependent manner. Plasmids pYL21-LexA and pYL21-LexA-YlREP1 were transformed into wild-type and Yl*ngs1*Δ cells carrying the reporter plasmid pINA445-lexAop-P_YlLEU2_-lacZ. Similarly, plasmids pYL21-LexA and pYL21-LexA-YlNGS1 were transformed into wild-type and Yl*rep1*Δ cells carrying the reporter plasmid pINA445-lexAop-P_YlLEU2_-lacZ. Cells were grown in liquid YNB-Glucose + GlcNAc medium. Cell lysates were measured for β-galactosidase activity. Mean data ± standard deviation from three independent experiments done on different days were plotted. The unpaired two-tailed Student’s *t*-test was used to examine the statistical significance of the difference between two samples. Statistically significant differences are indicated by the asterisks (***, *P* < 0.001). NS, not statistically significant.

In *C. albicans*, CaRep1 recruits CaNgs1 to the promoters of GlcNAc-inducible genes (19). Similar to CaRep1 and CaNgs1, yeast two-hybrid analysis showed that YlRep1 and YlNgs1 also interact physically (Fig. 2A). Surprisingly, this interaction appears to be mediated by the C-terminus of YlRep1 rather than the N-terminus. This is unexpected, as a recent report showed that CaNgs1 binds to the N-terminus of CaRep1 (28). This discrepancy suggests that notable differences exist between YlRep1 and CaRep1.

To determine whether YlRep1 and YlNgs1 may have transcriptional activation activity, we monitored the ability of LexA-YlRep1 and LexA-YlNgs1 fusion proteins (containing the DNA-binding domain of LexA, a.a. 1-87) to influence the expression of a *lexAop-P_YlLEU2_-lacZ* reporter in *Y. lipolytica*. In medium containing both glucose and GlcNAc, LexA-YlRep1 increased reporter expression by 10.3-fold, and LexA-YlNgs1 increased reporter expression by 9.1-fold in wild-type cells (Fig. 2B), indicating that they activate gene expression. In contrast, LexA-YlRep1 failed to increase reporter expression in Yl*ngs1*Δ cells (Fig. 2B), indicating that YlRep1 depends on YlNgs1 for gene activation. Interestingly, LexA-YlNgs1 also failed to increase reporter expression in Yl*rep1*Δ cells (Fig. 2B), indicating that YlRep1 is also required for gene activation by YlNgs1. The dependency on YlRep1 is likely due to the requirement of GlcNAc binding to YlNgs1 to activate gene expression (19). GlcNAc may not accumulate efficiently in Yl*rep1*Δ cells. Alternatively, YlRep1 may contribute to gene activation through other mechanisms. These results suggest that YlRep1 and YlNgs1 are interdependent for transcriptional activation.

Together, our results suggest that YlRep1 and YlNgs1 interact physically and work together to activate gene expression.

### GlcNAc inhibits filamentation in *Y. lipolytica*

**Fig 3:**
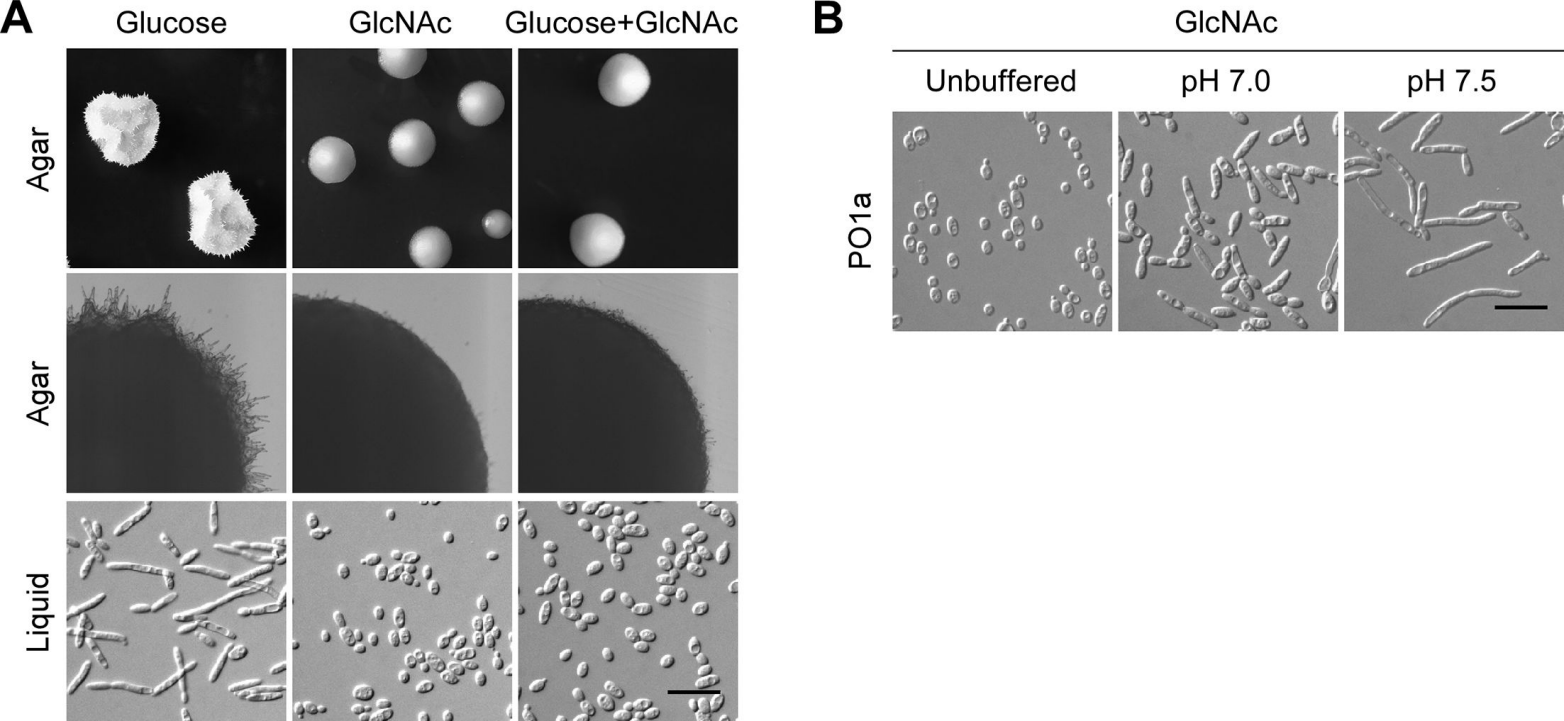
GlcNAc inhibits filamentation in *Y. lipolytica*. (**A**) GlcNAc inhibits filamentation. Wild-type strain PO1a was grown on YNB agar containing the indicated sugar for 3 days (upper row) or 2 days (middle row) or in liquid medium for 16 h (bottom row) at 30°C. (**B**) Alkaline pH induces filamentation in GlcNAc medium. Wild-type strain PO1a carrying pINA445 (Vec) was grown in liquid YNB-GlcNAc medium (unbuffered, pH ~5.4) or YNB-GlcNAc medium buffered to pH 7.0 or pH 7.5 with Na_2_HPO_4_-citric acid buffer for 16 h at 30°C. Bars, 20 µm. All the assays were done at least three times on different days.

GlcNAc is a strong inducer of filamentous growth in *C. albicans* (29). Based on this finding, GlcNAc has also been used previously to induce filamentation in *Y. lipolytica*, but often in combination with a citrate buffer at near-neutral pH (4, 30, 31). However, we found that GlcNAc alone is not a good inducer of filamentation. In contrast to glucose, which promotes filamentation in *Y. lipolytica*, GlcNAc inhibits filamentation as wild-type cells were in oval-shaped yeast form and did not elongate or form filaments in liquid or solid GlcNAc media as they did in glucose media (Fig. 3A). In addition, wild-type cells cultivated in liquid or solid media containing both glucose and GlcNAc displayed morphology identical to that of cells cultivated in GlcNAc media (Fig. 3A), indicating that GlcNAc can inhibit filamentation induced by glucose. The inhibition of filamentation by GlcNAc is not due to background mutations in the lab strain PO1a, as the wild-type strain W29 displayed the same cell morphology as strain PO1a under the same growth conditions (Fig. S1).

Although GlcNAc inhibits filamentation, alkaline pH can still induce filamentation in cells grown in GlcNAc medium (Fig. 3B). Wild-type cells grown in yeast nitrogen base (YNB)-GlcNAc medium buffered at pH 7.0 were elongated compared to those grown in unbuffered medium (pH ~5.4). 2% of cells (*n* > 200) grown in unbuffered medium were longer than 10 µm. In contrast, 65% and 7% of cells (*n* > 200) grown in GlcNAc medium buffered at pH 7.0 were longer than 10 and 20 µm, respectively. Cells grown in GlcNAc medium buffered at pH 7.5 formed long filaments, and 82% of cells (*n* > 200) were longer than 20 µm. This finding agrees with previous reports that filamentation can be induced in GlcNAc medium by alkaline pH and supports the idea that the “filament-inducing” effect of GlcNAc is due to neutral pH (see Discussion).

### GlcNAc inhibition of filamentation does not depend on the alteration of ambient pH

In *C. albicans*, in contrast to glucose catabolism, which decreases the pH of the culture medium, GlcNAc catabolism raises the pH of the medium, which indirectly induces filamentation (32). To investigate whether GlcNAc catabolism may also alter ambient pH in *Y. lipolytica*, we determined the initial pH of the culture medium and the final pH after 16 h of cultivation with the wild-type strain at 30°C. In both unbuffered media (pH ~5.4) and buffered pH 7.0 media with GlcNAc or glucose as the sole carbon source, there was a slight decrease in pH after the cultivation, and GlcNAc catabolism decreased ambient pH less than glucose catabolism (Table 1). Although GlcNAc did not induce filamentation in unbuffered medium and induced a low degree of filamentation in buffered pH 7.0 medium, glucose induced filamentation in both unbuffered and buffered pH 7.0 media (Table 1). This result suggests that the slight decrease in ambient pH by GlcNAc catabolism is unlikely to be the major cause of GlcNAc inhibition of filamentation. Therefore, the inhibition of filamentation by GlcNAc does not depend on the alteration of ambient pH in *Y. lipolytica*, which is different from *C. albicans*.

**TABLE 1:** GlcNAc catabolism causes a slight decrease of pH in the culture media^*a*^

Carbon source	Initial pH	Final pH	Decrease in pH	Percentage of cells longer than 20 µm
1% GlcNAc	5.36	4.93	0.43	0
1% glucose	5.47	4.81	0.66	28
1% GlcNAc	7.0 (Na_2_HPO_4_-citric acid buffer)	6.78	0.22	7
1% glucose	7.0 (Na_2_HPO_4_-citric acid buffer)	6.05	0.95	53

^*a*^ The wild-type strain PO1a carrying the empty vector pINA445 was grown in liquid YNB-GlcNAc or YNB-Glucose media supplemented with uracil for 16 h at 30°C. This assay was done three times on different days. Representative results are shown.

### YlRep1-YlNgs1 is important for the inhibition of filamentation by GlcNAc

**Fig 4:**
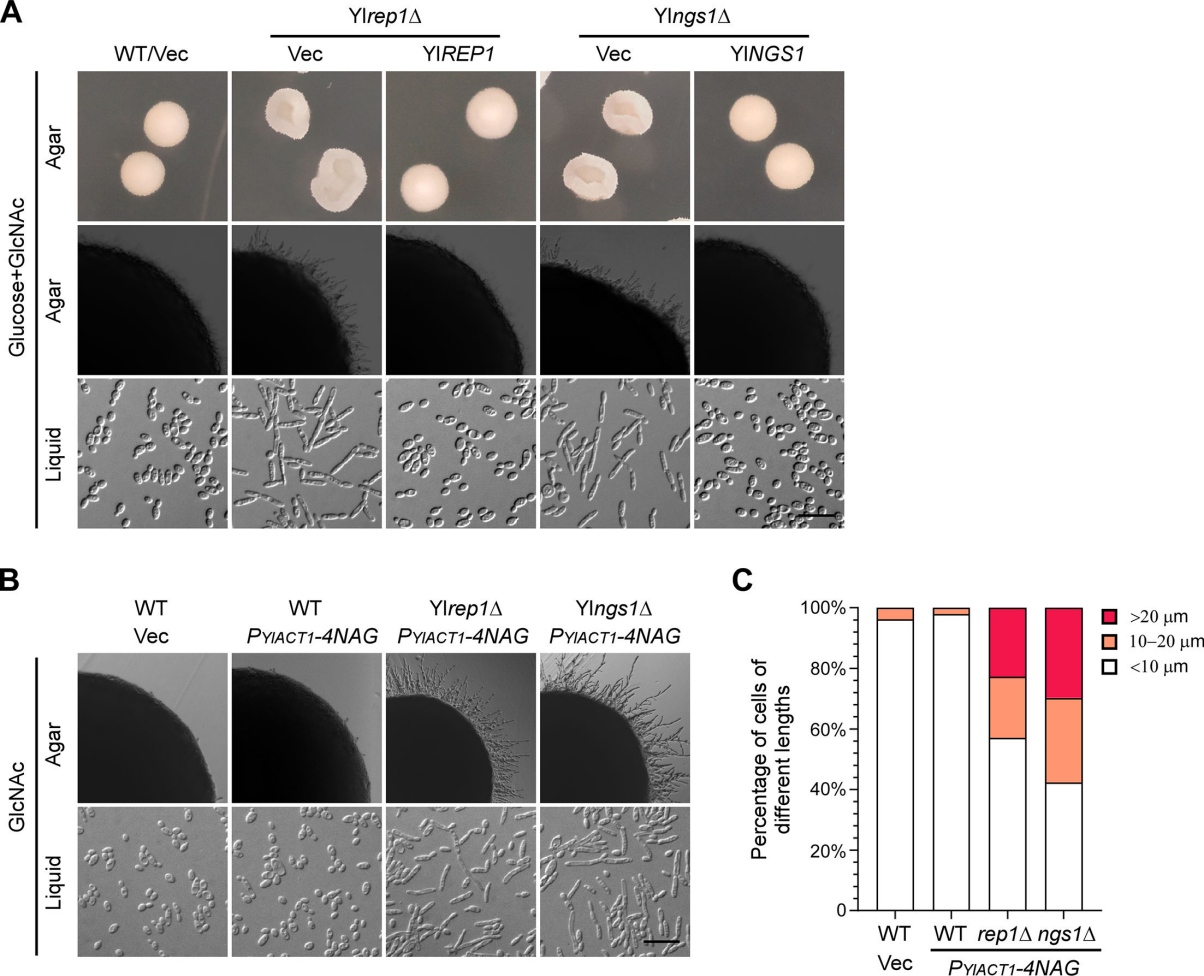
YlRep1 and YlNgs1 play a key role in the inhibition of filamentation by GlcNAc. (**A**) Morphology of Yl*rep1*Δ and Yl*ngs1*Δ colonies and cells grown in Glucose + GlcNAc medium. Wild-type (WT), Yl*rep1*Δ, and Yl*ngs1*Δ cells carrying pINA445 (Vec) or pINA445-Gene were grown on YNB-Glucose + GlcNAc agar for 3 days (upper row) or 2 days (middle row) or in liquid medium for 16 h (bottom row) at 30°C. (**B**) Yl*rep1*Δ and Yl*ngs1*Δ cells exhibited increased filamentation compared to wild-type cells in GlcNAc medium. Wild-type (WT), Yl*rep1*Δ, and Yl*ngs1*Δ cells carrying pYL27 (Vec) or pYL27-4NAG (*P_YlACT1_-4NAG*) were grown in liquid YNB-GlcNAc medium for 1 day at 30°C. Bar, 20 µm. (**C**) Percentage of cells of different lengths as in panel **B**. The cell lengths for each strain were categorized into three groups: <10 µm, 10–20 μm, and >20 µm. All the assays were done at least three times on different days. The data shown are representative of independent experiments.

Since YlRep1-YlNgs1 signaling is important for GlcNAc-induced expression of GlcNAc catabolic genes, we asked whether YlRep1-YlNgs1 is required for the inhibition of filamentation by GlcNAc. To this end, we examined the morphology of Yl*rep1*Δ and Yl*ngs1*Δ cells grown in media containing both glucose and GlcNAc. In contrast to wild-type cells that formed smooth colonies devoid of radial filaments on agar and were oval-shaped yeast form in liquid medium, Yl*rep1*Δ and Yl*ngs1*Δ cells formed wrinkled colonies with short radial filaments on agar and were elongated and formed filaments in liquid medium (Fig. 4A). This result suggests that both YlRep1 and YlNgs1 are required for the inhibition of filamentation by GlcNAc.

We also examined the morphology of wild-type, Yl*rep1*Δ, and Yl*ngs1*Δ cells carrying *P_YlACT1_-4NAG*, which grew in GlcNAc medium (Fig. 1E). On GlcNAc agar, wild-type cells carrying *P_YlACT1_-4NAG* or empty vector formed no radial filaments. In contrast, Yl*rep1*Δ cells and Yl*ngs1*Δ cells carrying *P_YlACT1_-4NAG* formed long radial filaments (Fig. 4B, upper panel). In liquid GlcNAc medium, wild-type cells carrying *P_YlACT1_-4NAG* or empty vector were in oval-shaped yeast form. Only 4% of wild-type cells carrying an empty vector and 2% of wild-type cells carrying *P_YlACT1_-4NAG* (*n* > 400) were in the range of 10–20 μm in length. None of them was longer than 20 µm. In contrast, Yl*rep1*Δ cells and Yl*ngs1*Δ cells carrying *P_YlACT1_-4NAG* formed short filaments. 20% of Yl*rep1*Δ cells carrying *P_YlACT1_-4NAG* and 28% of Yl*ngs1*Δ cells carrying *P_YlACT1_-4NAG* were in the range of 10–20 μm in length. Moreover, 23% of Yl*rep1*Δ cells carrying *P_YlACT1_-4NAG* and 30% of Yl*ngs1*Δ cells carrying *P_YlACT1_-4NAG* were longer than 20 µm (Fig. 4B and C). These results suggest that YlRep1 and YlNgs1 are important for the inhibition of filamentation by GlcNAc.

### YlRep1 and YlNgs1 repress a set of transcription factors and cell wall protein genes, some of which are associated with filamentation

To investigate how YlRep1 and YlNgs1 inhibit filamentation, we wanted to determine whether YlRep1 and YlNgs1 regulate the genes associated with filamentation. To this end, we conducted RNA-Seq analyses in wild-type, Yl*rep1*Δ, and Yl*ngs1*Δ cells carrying *P_YlACT1_-4NAG* grown in liquid GlcNAc medium. In Yl*rep1*Δ cells, 327 genes exhibited significant differential expression (≥2-fold, *P* < 0.05) compared to wild-type cells. Of these, 213 genes (65.1%) were upregulated, whereas 114 genes (34.9%) were downregulated (Table S1). In Yl*ngs1*Δ cells, 556 genes exhibited significant differential expression (≥2-fold, *P* < 0.05) compared to wild-type cells. Of these, 362 genes (65.1%) were upregulated, whereas 194 genes (34.9%) were downregulated in Yl*ngs1*Δ cells (Table S2). YlRep1 shares a large portion of target genes with YlNgs1. YlRep1 and YlNgs1 co-repress 188 genes, which account for 88.3% of YlRep1-repressed genes but only 51.9% of YlNgs1-repressed genes (Fig. S2). This result suggests that YlRep1 regulates gene expression mainly by recruiting YlNgs1 to target genes, whereas YlNgs1 can also be recruited to target genes by other transcription factors.

**Fig 5:**
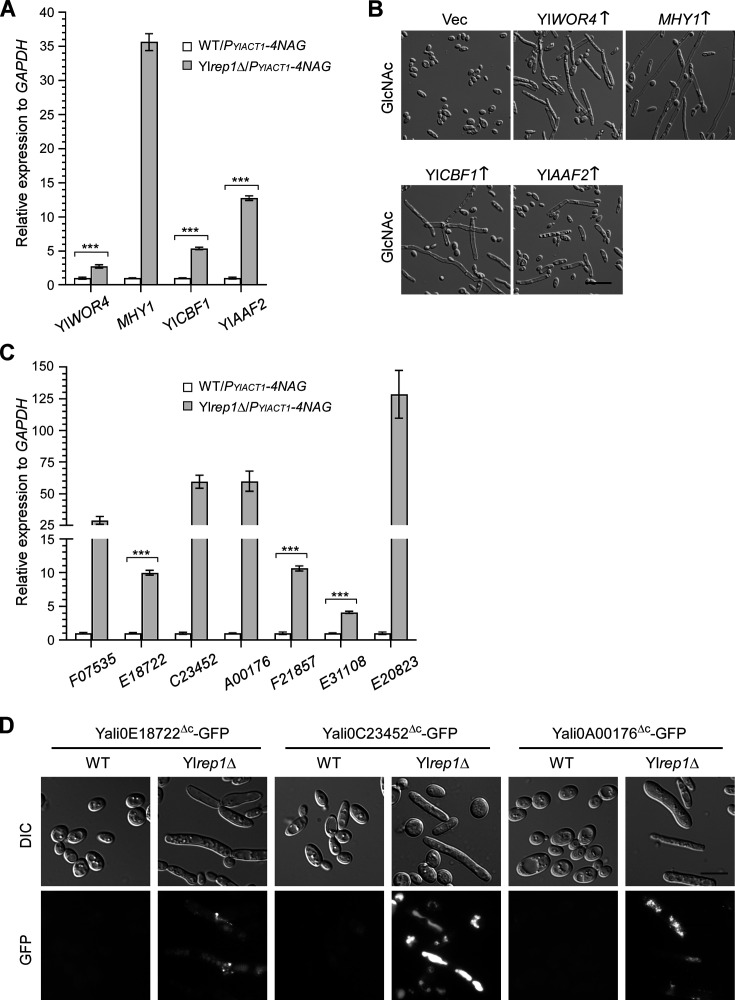
YlRep1 and YlNgs1 repress a set of transcription factors and cell wall protein genes, some of which are associated with filamentation. (**A**) The upregulation of Yl*WOR4*, *MHY1*, Yl*CBF1*, and Yl*AAF2* was validated by qRT-PCR. Wild-type (WT) and Yl*rep1*Δ cells carrying pYL27-4NAG (*P_YlACT1_-4NAG*) were grown in YNB-GlcNAc medium. The transcription levels of these genes were determined by qRT-PCR and normalized to *GAPDH*. (**B**) Overexpression of Yl*WOR4*, *MHY1*, Yl*CBF1*, or Yl*AAF2* caused filamentation in wild-type cells grown in GlcNAc medium. Wild-type cells carrying pYL13 (Vec), pYL13-YlWOR4, pYL13-MHY1, pYL13-YlCBF1, or pYL13-YlAAF2 were grown in liquid YNB-GlcNAc medium for 16 h at 30°C. The indicated genes were overexpressed under the control of the strong constitutive promoter of the Yl*TEF1* gene. Bar, 20 µm. (**C**) The upregulation of *YALI0F07535*, *YALI0E18722*, *YALI0C23452*, *YALI0A00176*, *YALI0F01857*, *YALI0E31108*, and *YALI0E20823* was validated by qRT-PCR. Wild-type (WT) and Yl*rep1*Δ cells carrying pYL27-4NAG (*P_YlACT1_-4NAG*) were grown in YNB-GlcNAc medium. The transcription levels of these genes were determined by qRT-PCR and normalized to *GAPDH*. (**D**) YlRep1 represses the adhesin-like genes *YALI0E18722*, *YALI0C23452*, and *YALI0A00176*. Wild-type (WT) and Yl*rep1*Δ cells carrying pYL25-4NAG (*P_YlACT1_-4NAG*) as well as pYL14-YALI0E18722^ΔC^, pYL14-YALI0C23452^ΔC^, or pYL14-YALI0A00176^ΔC^ were grown in YNB-GlcNAc medium and photographed for GFP fluorescence. Bar, 10 µm. The cell morphology and GFP fluorescence assays were done three times on different days. For the qRT-PCR assay, mean data ± standard deviation from three independent experiments done on different days were plotted. The unpaired two-tailed Student’s *t*-test was used to examine the statistical significance of the difference between two samples. Statistically significant differences are indicated by the asterisks (***, *P* < 0.001).

Transcription factors play important roles in regulating gene expression. YlRep1 repressed 17 transcription factor genes and highly repressed four of them (≥5-fold, *P* < 0.05) (Table 2). Fourteen of the 17 YlRep1-repressed genes (82.4%) are also repressed by YlNgs1 (Table 3). Among these genes, Yl*WOR4* (*YALI0F19822*), *MHY1*, Yl*CBF1* (*YALI0B3354*), Yl*AAF2* (*YALI0E06105*), and *YALI0E03410* were the top genes that exhibited the highest read counts. The upregulation of Yl*WOR4*, *MHY1*, Yl*CBF1*, and Yl*AAF2* was confirmed by quantitative reverse transcription PCR (qRT-PCR) analysis (Fig. 5A). While wild-type cells remained in the yeast form in liquid GlcNAc medium, the overexpression of Yl*WOR4*, *MHY1*, Yl*CBF1*, or Yl*AAF2* caused filamentation in wild-type cells (Fig. 5B). These findings suggest that YlRep1 may inhibit filamentation in part by repressing these transcription factor genes in GlcNAc medium.

**TABLE 2:** Upregulated and downregulated transcription factor and cell wall protein genes in Yl*rep1*Δ cells^*a*^

Direction of regulation	Transcription factor genes	Cell wall protein genes
Up (≥2-fold)	***WOR4* (*F19822***), ***F21923***, ***MOT3* (*E29271***), ***D14872***, *MHY1* (*B21582*), *E14971*, *CBF1* (*B13354*), *E03410, AAF2* (*E06105*), *F16511*, *NRG1* (*C12364*), *HOY1* (*A18469*), *C19151, PHD1* (*B19602*), *E02442*, *B13508, C02387*	***F07535***, ***E18722***, ***C23452***, ***A00176***, ***F21857***, ***E31108***, ***E20823***, *F00990*, *A20438*, *C14630*, *C15004*, *F22847*, *D00154*, *E01210*, *F21428*
Down (≥2-fold)	*C17017, F20152, D24860*	***B20174***, *D10967*, *F25773*, *A21373*, *F24255*, *D22957*, *A13013*, *E11715*, *D27214*

^*a*^ For simplicity, “*YALI0*” in the systematic name of each gene was omitted; for example, “*F19822*” stands for “*YALI0F19822.*” Genes that are highly differentially expressed (≥5-fold) are in bold. Genes that encode putative cell surface adhesins are underlined.

**TABLE 3:** The transcription factor and cell wall protein genes repressed by YlRep1 and YlNgs1 overlap substantially^*a*^

Genes	YlNgs1 repressed only	YlNgs1-YlRep1 co-repressed	YlRep1 repressed only
Transcription factor genes	*F03157*, *AAF1* (*E23925*), *F30261*, *MSN4* (*C13750*), *D10285*, *B18942*, *D12628*, *E02178*, *E12023*	***WOR4* (*F19822***)^n,r^, ***MOT3* (*E29271***)^n,r^, ***F21923***^n,r^, ***D14872***^n,r^, ***E03410***^n^, ***MHY1* (*B21582***)^n^, ***CBF1* (*B13354***)^n^, *F16511*, *E14971*, *AAF2* (*E06105*), *HOY1* (*A18469*), *B13508*, *E02442*, *NRG1* (*C12364*)	*C19151*, *PHD1* (*B19602*), *C02387*
Cell wall protein genes	***A15378***, *D09185*, *F10549*, *F26565*, *D25938*, *D24277*, *E05819*, *B18194*, *D24101*, *F01925*, *D14300*, *A17919*, *E28534*	***F07535***^n,r^, ***E18722***^n,r^, ***C23452***^n,r^, ***A00176***^n,r^, ***F21857***^n,r^, ***E31108***^n,r^, ***E20823***^n,r^, ***F00990***^n^, *A20438*, *C14630*, *C15004*, *F22847*, *D00154*, *E01210*, *F21428*	None

^*a*^ Genes that displayed highly differential expression (≥5-fold) in Yl*rep1*Δ or Yl*ngs1*Δ cells carrying *P_YlACT1_-4NAG* are in bold. For YlRep1-YlNgs1-co-repressed genes, the superscripts “r” and “n” indicate highly differentially expressed genes in Yl*rep1*Δ (r) or Yl*ngs1*Δ (n) cells. Genes that encode putative cell surface adhesins are underlined.

During the yeast-to-filament transition, the cell wall undergoes extensive reorganization to support hyphal growth. This process is accompanied by the upregulation and downregulation of some cell wall protein genes. RNA-Seq analysis revealed that, among the cell wall protein genes, 15 genes were upregulated (≥2-fold, *P* < 0.05), whereas nine genes were downregulated in Yl*rep1*Δ cells (Table 2). Of these, seven genes were highly upregulated (≥5-fold, *P* < 0.05), whereas only one gene was highly downregulated. Interestingly, all the 15 YlRep1-repressed cell wall protein genes are also repressed by YlNgs1 (Table 3), suggesting that they are co-repressed by YlRep1 and YlNgs1. We measured the expression of seven highly upregulated genes by qRT-PCR. Consistent with the RNA-Seq data, all seven genes were markedly upregulated in Yl*rep1*Δ cells (Fig. 5C). Among the seven highly upregulated genes, *YALI0F07535* and *YALI0E31108* were upregulated by more than 10-fold in Yl*rep1*Δ cells. They encode proteins similar to *S. cerevisiae* structural cell wall proteins Tir3 and *Y. lipolytica* cell wall protein Cwp1, respectively. These genes may be the primary targets of YlRep1 repression.

Among the seven highly upregulated cell wall protein genes, *YALI0E18722*, *YALI0C23452*, and *YALI0A00176* encode proteins similar to *S. cerevisiae* adhesin Flo11 (Table 2). The three adhesin-like genes were upregulated by more than 15-fold and displayed high read counts in Yl*rep1*Δ cells. *YALI0C23452* and *YALI0A00176* are also highly upregulated during alkaline-induced filamentation (33). We tagged the three adhesin-like genes at the 3′-terminus of ORF with GFP (the short C-terminus containing the GPI modification site was deleted from each protein) and detected these proteins in live cells. Yl*rep1*Δ cells carrying three GFP-tagged genes all exhibited brighter GFP fluorescence than wild-type cells carrying the same construct (Fig. 5D). This result suggests that the three adhesin-like genes are targets of YlRep1 repression. For unclear reasons, the GFP fluorescence was predominantly retained intracellularly instead of being localized on the cell surface. This was likely due to the recycling of cell surface proteins into the vacuoles.

It should be noted that Yl*rep1*Δ and Yl*ngs1*Δ cells carrying *P_YlACT1_-4NAG* grew slower than wild-type cells carrying *P_YlACT1_-4NAG* or empty vector in liquid GlcNAc medium. This may have an impact on gene expression. Together, our results suggest that YlRep1 and YlNgs1 co-repress a set of transcription factors and cell wall protein genes, some of which are associated with filamentation.

### GlcNAc catabolism is not required for GlcNAc inhibition of filamentation

**Fig 6:**
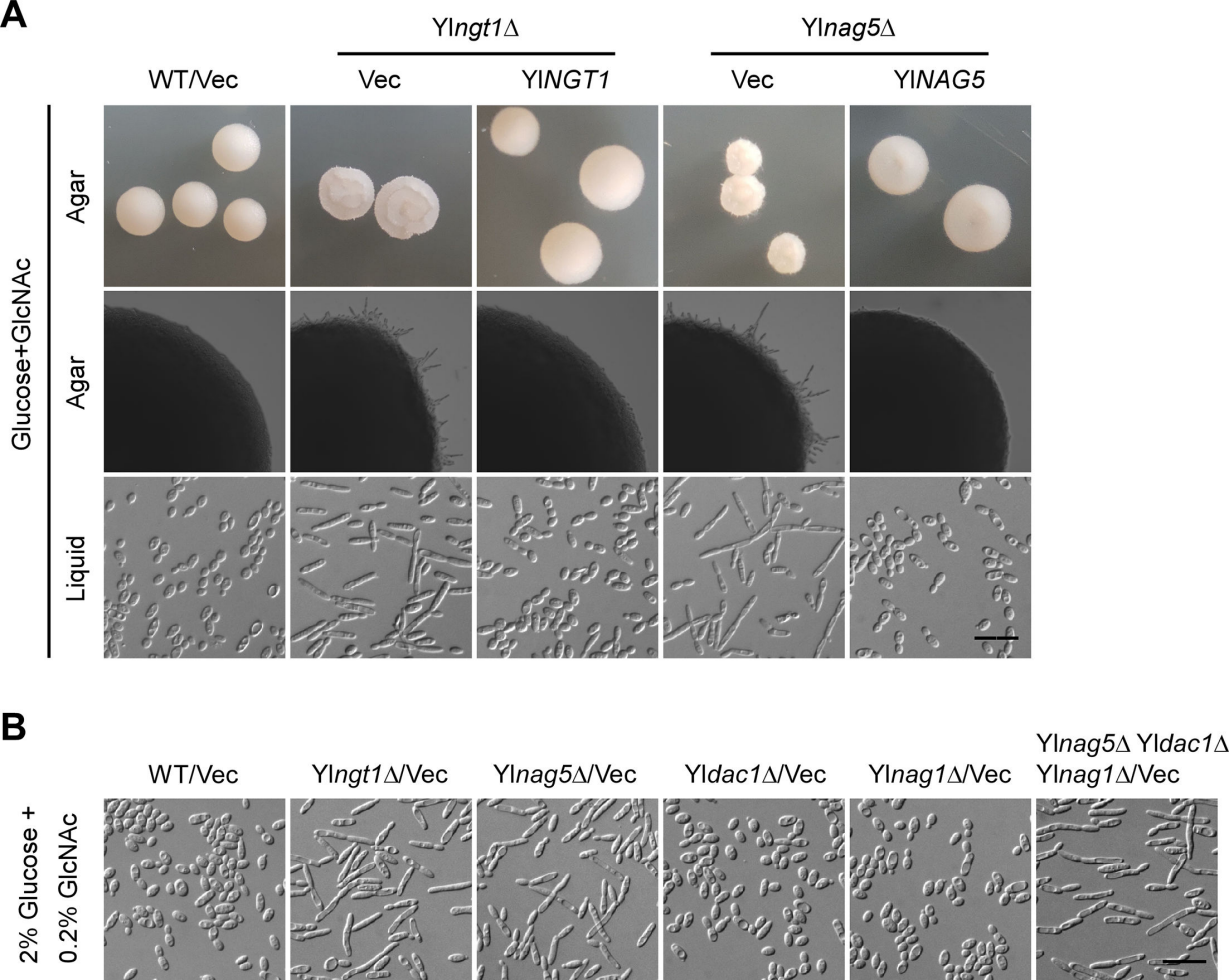
GlcNAc catabolism is not required for the inhibition of filamentation by GlcNAc. (**A**) Colony and cell morphologies of Yl*ngt1*Δ and Yl*nag5*Δ strains in Glucose + GlcNAc medium. Wild-type (WT), Yl*ngt1*Δ, and Yl*nag5*Δ cells carrying pINA445 (Vec) or pINA445-Gene were grown on YNB-Glucose + GlcNAc agar for 3 days (upper row) or 2 days (middle row) or in liquid medium for 16 h (bottom row) at 30°C. (**B**) GlcNAc inhibits filamentation in Yl*dac1*Δ and Yl*nag1*Δ cells. Wild-type (WT), Yl*ngt1*Δ, Yl*nag5*Δ, and Yl*nag5*Δ Yl*dac1*Δ Yl*nag1*Δ cells carrying pINA445 (Vec) were grown in liquid YNB-2% Glucose + 0.2% GlcNAc medium for 16 h at 30°C. Yl*dac1*Δ and Yl*nag1*Δ cells carrying pINA445 were grown in the same medium until their cell densities were comparable to that of wild-type cells (~24 h). All the assays were done at least three times on different days. Bars, 20 µm.

In *C. albicans*, Ca*hxk1*Δ and Ca*hxk1*Δ Ca*dac1*Δ Ca*nag1*Δ cells are unable to catabolize GlcNAc but can still be induced to form hyphae, suggesting that GlcNAc catabolism is not required for the induction of filamentation by GlcNAc (27). To determine whether GlcNAc catabolism is required for the inhibition of filamentation by GlcNAc in *Y. lipolytica*, we examined the morphology of Yl*ngt1*Δ and Yl*nag5*Δ cells (YlNag5 is the ortholog of *C. albicans* CaHxk1) grown in medium containing both glucose and GlcNAc (note: Yl*dac1*Δ and Yl*nag1*Δ cells did not grow in this medium). In contrast to wild-type cells that did not form any filaments, Yl*ngt1*Δ and Yl*nag5*Δ cells formed wrinkled colonies with short radial filaments on solid medium and were elongated and formed filaments in liquid media (Fig. 6A). YlNgt1 is the GlcNAc transporter on the cell surface. Since Yl*ngt1*Δ cells are strongly defective in growth in GlcNAc medium (Fig. 1B), the growth seen here is most likely supported by glucose. This result suggests that GlcNAc needs to enter the cells efficiently to inhibit filamentation. The observation that Yl*nag5*Δ cells still formed filaments in this medium suggests that GlcNAc catabolism might be required for the inhibition of filamentation by GlcNAc.

To make sure that this idea is correct, we wanted to examine the morphology of Yl*dac1*Δ and Yl*nag1*Δ cells, which are also unable to catabolize GlcNAc. To solve the problem of growth inhibition by GlcNAc, we cultured Yl*dac1*Δ and Yl*nag1*Δ cells continuously in liquid medium containing both glucose and GlcNAc to isolate growing cells. The concentration of GlcNAc was initially set at 0.05% and gradually increased to 0.2%. We eventually isolated Yl*dac1*Δ and Yl*nag1*Δ cells that grew in medium containing both 2% glucose and 0.2% GlcNAc. Interestingly, Yl*dac1*Δ and Yl*nag1*Δ cells were in the oval-shaped yeast form. None of the Yl*dac1*Δ and Yl*nag1*Δ cells were longer than 20 µm. In contrast, Yl*ngt1*Δ, Yl*nag5*Δ, and Yl*nag5*Δ Yl*dac1*Δ Yl*nag1*Δ cells were elongated and formed filaments (Fig. 6B). Twenty-nine percent of Yl*ngt1*Δ, 25% of Yl*nag5*Δ, and 27% of Yl*nag5*Δ Yl*dac1*Δ Yl*nag1*Δ cells (*n* > 400) grown in this medium were longer than 20 µm. This result suggests that the inhibition of filamentation by GlcNAc requires YlNag5 but not GlcNAc catabolism.

### The transcriptional repression of YlRep1 may require YlNag5

**Fig 7:**
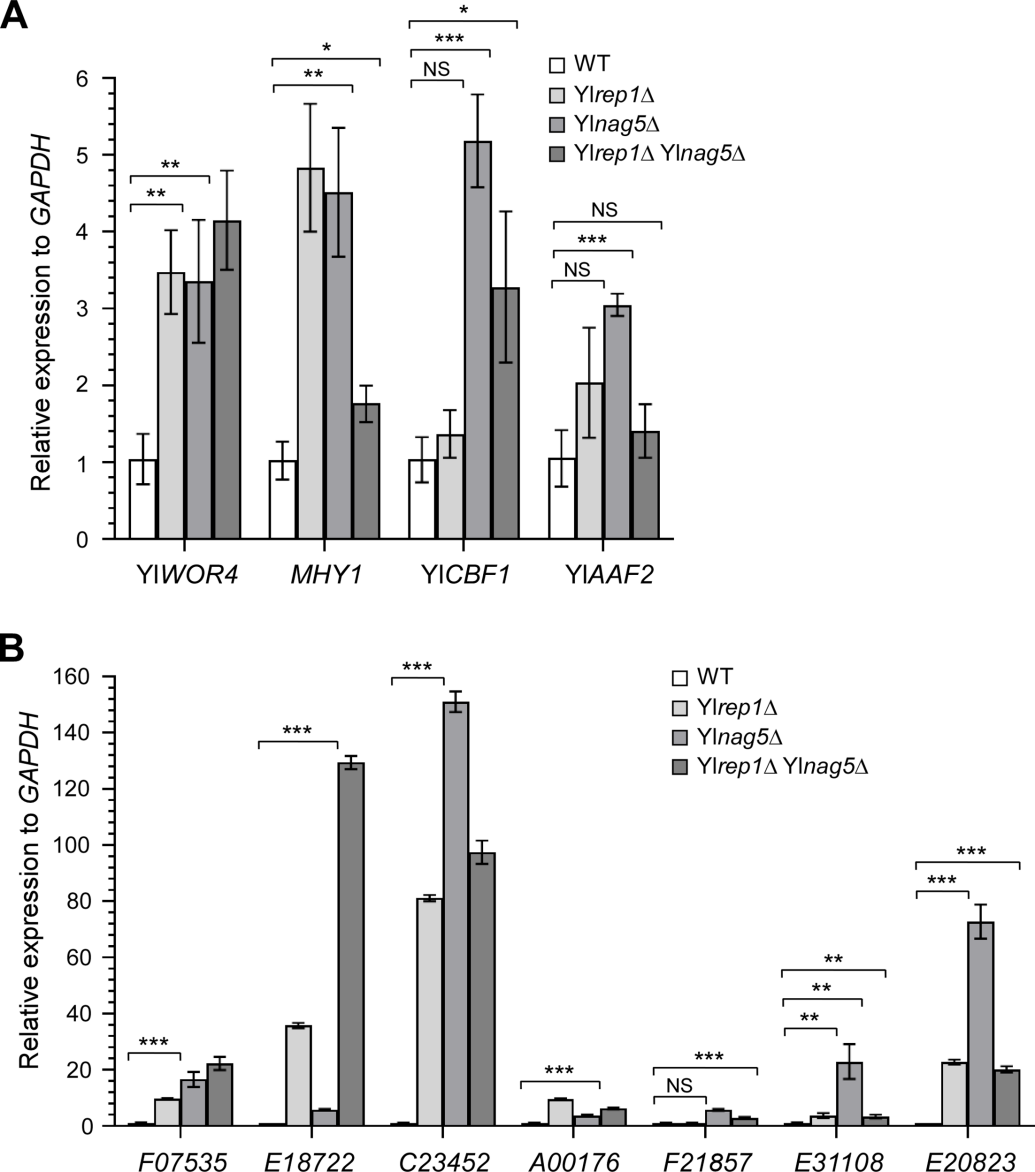
The transcriptional repression of YlRep1 may require YlNag5. The major transcription factor and cell wall protein genes repressed by YlRep1 were upregulated in Yl*nag5*Δ cells. The transcription levels of transcription factor genes Yl*WOR4*, *MHY1*, Yl*CBF1*, Yl*AAF2* (**A**), and cell wall protein genes *YALI0F07535*, *YALI0E18722*, *YALI0C23452*, *YALI0A00176*, *YALI0F01857*, *YALI0E31108*, and *YALI0E20823* (**B**) in wild-type (WT), Yl*rep1*Δ, Yl*nag5*Δ, and Yl*rep1*Δ Yl*nag5*Δ cells grown in YNB-Glucose + GlcNAc medium were determined by qRT-PCR and normalized to *GAPDH*. All qRT-PCR data showed mean ± standard deviation from three independent experiments done on different days. The unpaired two-tailed Student’s *t*-test was used to examine the statistical significance of the difference between two samples. Statistically significant differences are indicated by the asterisks (*, *P* < 0.05; **, *P* < 0.01; ***, *P* < 0.001). NS, not statistically significant.

Because Yl*rep1*Δ cells and Yl*nag5*Δ cells formed filaments in liquid medium containing both glucose and GlcNAc (Fig. 4A and 6A), this raises the possibility that YlNag5 may be required for YlRep1 to repress filamentation-related genes. To explore this possibility, we measured the expression levels of major YlRep1-repressed transcription factor and cell wall protein genes (identified by RNA-Seq) in wild-type, Yl*rep1*Δ, Yl*nag5*Δ, and Yl*rep1*Δ Yl*nag5*Δ cells grown in medium containing both glucose and GlcNAc (note: this medium is different from the GlcNAc medium used for RNA-Seq). For the four transcription factor genes, *MHY1* and Yl*WOR4* were markedly upregulated in Yl*rep1*Δ cells grown in this medium. They were also significantly upregulated in Yl*nag5*Δ and Yl*rep1*Δ Yl*nag5*Δ cells (Fig. 7A). Although Yl*AAF2* and Yl*CBF1* were not significantly upregulated in Yl*rep1*Δ cells grown in this medium, they were significantly upregulated in Yl*nag5*Δ cells, and Yl*CBF1* was also significantly upregulated in Yl*rep1*Δ Yl*nag5*Δ cells (Fig. 7A). For the seven cell wall protein genes, all of them were significantly upregulated in Yl*rep1*Δ cells except *YALI0F21857*, and they all were significantly upregulated in Yl*nag5*Δ and Yl*rep1*Δ Yl*nag5*Δ cells (Fig. 7B). These results indicate that major YlRep1-repressed transcription factor and cell wall protein genes are all upregulated in Yl*nag5*Δ cells grown in medium containing both glucose and GlcNAc, suggesting that the repression of these genes by YlRep1 may require YlNag5.

## DISCUSSION

GlcNAc is a common source of carbon and nitrogen in nature, present in both terrestrial and aquatic environments, as well as in animal intestines. Surprisingly, it is also a signaling molecule that stimulates morphogenetic transitions in fungi. In this study, we show that GlcNAc inhibits filamentation in the dimorphic yeast *Y. lipolytica*. The Rep1-Ngs1 complex not only regulates the induction of GlcNAc catabolic genes but also the inhibition of filamentation by GlcNAc. GlcNAc inhibition of filamentation requires the GlcNAc kinase YlNag5 but operates independently of GlcNAc catabolism. These results provide new insights into the GlcNAc-regulated dimorphic transition in *Y. lipolytica*.

GlcNAc is a potent inducer of filamentation in *C. albicans* (29). Based on this observation, researchers studying dimorphic transition in *Y. lipolytica* also tested GlcNAc for its ability to induce filamentation, often using it in combination with a citrate buffer at near-neutral pH (pH 6–7) (see a summary in Table S3) (4, 30, 31). Sometimes, additional treatment of the cells, such as brief cold treatment, was also employed to increase the efficiency (4, 31). Since this combination proved effective, many papers that cited the results have described GlcNAc as an inducer of filamentation in *Y. lipolytica*. However, because citrate and particularly neutral pH are strong inducers of filamentation (5), the exact role of GlcNAc in regulating filamentation in *Y. lipolytica* remains unclear. One study reported that GlcNAc alone induces filamentation poorly compared to glucose and suggested that the apparent “filament-inducing” effect of GlcNAc might actually be due to neutral pH and citrate (see Table S3) (5). Our findings support this idea. While GlcNAc inhibits filamentation, we observed that cells grown in GlcNAc were still able to form filaments in response to alkaline pH induction, in agreement with previous reports. Similarly, GlcNAc has been shown to strongly inhibit filamentation in *C. tropicalis* (25), which is closely related to *C. albicans*. However, the mechanism underlying this inhibition is not clear.

In *C. albicans*, GlcNAc induces filamentation via both alkalization of the culture medium and CaNgs1 signaling (19, 21, 32). In contrast, GlcNAc inhibits filamentation primarily via YlRep1-YlNgs1 signaling in *Y. lipolytica*. Although GlcNAc catabolism slightly decreases ambient pH, it is less strong than glucose catabolism. Based on the observation that glucose induces filamentation even though glucose catabolism decreases ambient pH, the decrease of ambient pH by GlcNAc catabolism is not critical for the inhibition of filamentation.

Why does GlcNAc promote filamentation in *C. albicans* but inhibit filamentation in *Y. lipolytica* and *C. tropicalis*? These opposing, species-specific responses likely reflect the distinct ecological niches and evolutionary pressures faced by these species. *C. albicans*, which typically resides within the human body, uses filamentation as a strategy to invade host tissues more effectively and evade immune responses (8). Since GlcNAc is naturally present in the host environment as a component of the extracellular matrix of host cells and a cell wall component of commensal microbiota, it likely acts as a signal to *C. albicans* that it is in a host environment, triggering filamentation as a survival and invasive strategy. In contrast, *Y. lipolytica* and *C. tropicalis* primarily inhabit soil and water (34–36). GlcNAc may act more as a nutrient source than a morphogenic signal for filamentation in *Y. lipolytica* and *C. tropicalis*.

The Ndt80 family transcription factor Rep1 (called Ron1 in filamentous fungi) is essential for GlcNAc utilization and the induction of GlcNAc catabolic genes in yeast species *C. albicans*, *C. tropicalis*, and *Y. lipolytica,* as well as in filamentous fungi, such as *T. reesei* (19, 23, 26). The histone acetyltransferase Ngs1 is widely conserved across fungal species that can metabolize GlcNAc, where Ngs1 functions as the GlcNAc sensor and transducer (19, 24). Several studies, including this one, have shown that the Rep1-Ngs1 complex plays a critical role in inducing GlcNAc catabolic genes in various fungal species (19, 24, 37). These results suggest that the role of Rep1-Ngs1 in regulating GlcNAc catabolic genes might be evolutionarily conserved.

In *C. albicans*, the induction of GlcNAc catabolic genes requires CaRep1-CaNgs1 signaling, whereas GlcNAc-induced filamentation requires CaNgs1 but not CaRep1 (19, 21). CaNgs1 is thought to bind to the promoter of the transcription factor CaBrg1 and induce its expression in the presence of GlcNAc. CaBrg1, in turn, downregulates the expression of CaNrg1, a major repressor of filamentation, leading to hyphal development (19). Notably, this regulation is independent of GlcNAc catabolism. In the human pathogen *C. tropicalis*, GlcNAc strongly inhibits filamentation (25), which is similar to the regulation observed in *Y. lipolytica*. Interestingly, CtRep1 is also required for the inhibition of filamentation by GlcNAc, as filamentation occurred in Ct*rep1*Δ cells but not in wild-type cells grown in medium containing both glucose and GlcNAc (26). This finding closely parallels our observations in *Y. lipolytica*. In this study, we demonstrate that both YlRep1 and YlNgs1 are essential for GlcNAc inhibition of filamentation in *Y. lipolytica*. YlRep1 likely functions by recruiting YlNgs1 to the promoters of target genes. Together, they repress filamentation-related genes, including the key regulator of filamentation *MHY1* (which encodes a transcription factor) and three adhesin-like genes.

Our results indicate that the YlRep1-YlNgs1 complex plays dual and opposing roles in regulating gene expression. In response to GlcNAc, it activates GlcNAc catabolic genes but represses filamentation-related genes. In *C. tropicalis*, where GlcNAc also inhibits filamentation, although it is not yet known about CtNgs1’s role, CtRep1 also activates GlcNAc catabolic genes but inhibits filamentation (26), which resembles *Y. lipolytica* YlRep1. In *C. albicans*, where GlcNAc induces filamentation, although CaRep1 is not involved in regulating GlcNAc-induced filamentation, CaRep1 is initially reported to repress the expression of *MDR1* efflux pump involved in drug resistance (20), indicating that CaRep1 also plays dual and opposing roles in regulating gene expression. In this study, we show that YlRep1 and YlNgs1 exhibit transcriptional activation activity, which could explain how the Rep1-Ngs1 complex activates GlcNAc catabolic genes in response to GlcNAc. However, it remains unclear how YlRep1-YlNgs1 represses gene expression.

In this study, we observed that GlcNAc failed to inhibit filamentation in Yl*nag5*Δ and Yl*nag5*Δ Yl*dac1*Δ Yl*nag1*Δ cells grown in medium containing both glucose and GlcNAc, suggesting that YlNag5 is important for the inhibition of filamentation by GlcNAc. Supporting this finding, we observed that major transcription factor and cell wall protein genes repressed by YlRep1 were upregulated in Yl*nag5*Δ cells grown in medium containing both glucose and GlcNAc, indicating that YlNag5 may be required for YlRep1 to repress filamentation-related genes. However, the exact mechanism underlying this YlNag5 function is unclear.

In *C. tropicalis*, where GlcNAc also inhibits filamentation, GlcNAc still inhibited filamentation in Ct*hxk1*Δ cells (CtHxk1 is the ortholog of YlNag5) while CtHxk1 overexpression drastically increased filamentous growth on Lee’s media containing GlcNAc (38), suggesting that CtHxk1 promotes filamentation rather than inhibiting it. Interestingly, a previous report indicated that overexpression of YlNag5 led to strong filamentation in *Y. lipolytica* cells grown in media containing glucose, glycerol, or GlcNAc (14), suggesting that YlNag5 may function similarly to CtHxk1. However, in our study, we did not observe a similar effect when YlNag5 was overexpressed.

Our study characterizes the inhibitory effect of GlcNAc on filamentous growth in *Y. lipolytica* and identifies YlRep1-YlNgs1 as a key regulator in the inhibition of filamentation by GlcNAc. It also suggests that the repression of filamentation-related genes by YlRep1 may require the GlcNAc kinase YlNag5. However, there are also limitations in this study. For example, some of the mutants generated in this study, such as Yl*rep1*Δ and Yl*ngs1*Δ cells carrying *P_YlACT1_-4NAG,* grew slower than wild-type cells carrying *P_YlACT1_-4NAG* or empty vector in liquid GlcNAc medium. Moreover, Yl*dac1*Δ and Yl*nag1*Δ cells also grew slower than wild-type cells in medium containing 2% glucose and 0.2% GlcNAc. The slow-growth phenotype of these mutants may have an impact on gene expression and filamentation. We observed that YlRep1-YlNgs1 exhibits opposing effects on filamentation compared to *C. albicans* CaNgs1. It is not clear what factor may determine the divergent outcomes between these two yeast species. It is also not clear why GlcNAc fails to inhibit filamentation in Yl*nag5*Δ and Yl*nag5*Δ Yl*dac1*Δ Yl*nag1*Δ cells. Future investigations are needed to address these questions.

## MATERIALS AND METHODS

### Strains and media

The *Y. lipolytica* strains used in this study are listed in Table S4 in the supplemental material. PO1a (*MATA leu2-270 ura3-302*) was used as the wild-type strain. *Y. lipolytica* strains were grown at 30°C. Culture media include yeast extract-peptone-dextrose (YPD) medium (20 g/L peptone, 10 g/L yeast extract, 2% glucose) and YNB medium (6.7 g/L yeast nitrogen base without amino acid) containing GlcNAc (1%), glucose (1%), glycerol (1%), or glucose + GlcNAc (1% glucose, 1% GlcNAc) supplemented with 80 mg/L leucine, 20 mg/L uracil, or both, when required. Na_2_HPO_4_-citric acid buffer was used to adjust the medium to pH 7.0 or pH 7.5 when required. *S. cerevisiae* strains used are listed in Table S4. *E. coli* strain DH5α was used for plasmid amplification.

### Plasmid construction

The plasmids used in this study are listed in Table S5. The oligonucleotide primers are listed in Table S6. To generate pINA445-YlREP1, Yl*REP1* carrying a 2,000 bp promoter and 400 bp 3′-UTR was amplified from genomic DNA by PCR and inserted into *Hin*dIII-digested vector pINA445 (*CEN*, *LEU2*) using ClonExpress II One Step Cloning Kit (Vazyme Biotech Co., China). Similarly, Yl*NGS1* (carrying 1,400 bp promoter and 400 bp 3′-UTR), Yl*NDT80* (carrying 500 bp promoter and 500 bp 3′-UTR), Yl*NGT1* (carrying 2,000 bp promoter and 500 bp 3′-UTR), Yl*NAG5* (carrying 841 bp promoter and 500 bp 3′-UTR), Yl*DAC1* (carrying 2,000 bp promoter and 517 bp 3′-UTR), and Yl*NAG1* (carrying 2,000 bp promoter and 500 bp 3′-UTR) were inserted into pINA445, yielding pINA445-YlNGS1, pINA445-YlNDT80, pINA445-YlNGT1, pINA445-YlNAG5, pINA445-YlDAC1, and pINA445-YlNAG1, respectively.

To generate the plasmid that carries four GlcNAc catabolic genes (NAG) under the control of Yl*ACT1* promoter, the 947 bp Yl*ACT1* promoter (nucleotides −950 to −4 relative to the first nucleotide in the start codon) was amplified by PCR from genomic DNA and fused to the ORFs of four GlcNAc catabolic genes by overlapping PCR. The resulting expression cassettes *P_YlACT1_*-Yl*NGT1*, *P_YlACT1_*-Yl*NAG5*, *P_YlACT1_*-Yl*DAC1*, and *P_YlACT1_*-Yl*NAG1* were inserted into pYL25 (*CEN*, Yl*URA3*) and pYL27 (*CEN*, Yl*LEU2*), yielding pYL25-4NAG and pYL27-4NAG, respectively.

To monitor the transcriptional activities of Yl*NGT1*, Yl*NAG5*, Yl*DAC1*, and Yl*NAG1* promoters in Yl*rep1*Δ cells and Yl*ngs1*Δ cells versus wild-type cells, the promoter region of each gene plus the ATG start codon was amplified by PCR and inserted into pINA445-*lacZ* using the ClonExpress II One-Step Cloning Kit, yielding pINA445-P_YlNGT1_-*lacZ*, pINA445-P_YlNAG5_-*lacZ*, pINA445-P_YlDAC1_-*lacZ*, and pINA445-P_YlNAG1_-*lacZ*.

To generate LexA-YlRep1 and LexA-YlNgs1 for one-hybrid assay, the DNA-binding domain (a.a. 1-87) of the *E. coli lexA* gene was amplified and inserted into *Hin*dIII-digested pYL21, yielding pYL21-lexA. Similarly, *lexA-YlREP1* and *lexA-YlNGS1* were generated by overlapping PCR and inserted into pYL21, yielding pYL21-lexA-YlREP1 and pYL21-lexA-YlNGS1. pINA445-lexAop-P_YlLEU2_-lacZ was reported previously (39).

To overexpress the transcription factor genes, the ORFs of Yl*WOR4*, Yl*CBF1*, *MHY1*, and Yl*AAF2* plus 300 bp 3′-UTR were amplified by PCR and inserted into pYL13 (*CEN*, Yl*LEU2*, *P_YlTEF1_*), yielding pYL13-YlWOR4, pYL13-YlCBF1, pYL13-MHY1, and pYL13-YlAAF2, respectively.

To visualize the transcriptional activities of the adhesin-like genes that were highly repressed by YlRep1, *YALI0C23452^1-794^* carrying a 2,674 bp promoter, *YALI0E18722^1-608^* carrying a 1,775 bp promoter, and *YALI0A00176^1-825^* carrying a 2,000 bp promoter were amplified by PCR and inserted into *Bam*HI-digested pYL14, yielding pYL14-YALI0C23452^ΔC^, pYL14-YALI0E18722^ΔC^, and pYL14-YALI0A00176^ΔC^, respectively.

For the two-hybrid assay, full-length Yl*NGS1* ORF was amplified from genomic DNA by PCR and inserted into *Bam*HI-digested pGAD-C1 (2μ, *LEU2*, *GAL4-AD*) and pGBDU-C1 (2μ, *URA3*, *GAL4-DBD*), yielding pGAD-YlNGS1 and pGBDU-YlNGS1, respectively. Similarly, full-length Yl*REP1*, Yl*REP1^1-200^*, Yl*REP1^1-376^*, and Yl*REP1^377-494^* segments were amplified from genomic DNA by PCR and inserted into *Bam*HI-digested pGAD-C1 and pGBDU-C1, yielding pGAD-YlREP1, pGAD-YlREP1 segments, pGBDU-YlREP1 and pGBDU-YlREP1 segments.

### Yeast strain construction

Yl*NGT1*, Yl*NAG5*, Yl*DAC1*, Yl*NAG1*, Yl*REP1*, Yl*NGS1*, and Yl*NDT80* were deleted in *Y. lipolytica* strains by homologous recombination following standard procedure (39).

### β-Galactosidase assay

The β-galactosidase activity in the cells was determined by the crude cell extract assay using O-nitrophenyl-β-D-galactopyranoside (ONPG) as the substrate, as reported previously (39).

### Yeast two-hybrid assay

pGAD-C1-based plasmids were transformed into the *S. cerevisiae* haploid strain pJ69-4A. pGBDU-C1-based plasmids were transformed into the haploid strain pJ69-4α. Pairs of haploid strains were mated on YPD plates and then replica plated onto SC-Leu-Ura plates to select for diploid cells that harbor both bait and prey plasmids. Diploid cells were patched on an SC-Leu-Ura plate and replica plated onto an SC-Leu-Ura-Ade plate to check for growth. Growth indicates interaction between the DNA-binding domain (DBD) and activation domain (AD) fusion proteins.

### RNA-Seq analysis

*Y. lipolytica* cells were grown in liquid YNB-GlcNAc medium supplemented with uracil at 30°C and collected at an OD600 of ~0.8. Each sample was in triplicate. RNA-Seq was conducted at Personalbio (Shanghai, China). Differential expression analysis between two conditions was performed on Personalbio Genescloud. Differentially expressed genes were defined as those for which the adjusted *P* value <0.05 and the fold change ≥2.0.

### RNA extraction and quantitative real-time PCR analysis

Yeast cells grown in 50 mL of culture at 30°C were collected at an OD_600_ of ~0.8. Total RNAs were extracted using Yeast RNA Kit (Omega, China). RNA integrity was examined by 1% agarose gel electrophoresis. The yields of RNAs were examined by the NanoDrop One Spectrometer. One microgram total RNA per sample was used for reverse transcription using the HiScript II Q RT SuperMix for qPCR (Vazyme Biotech Co., China). qPCRs were carried out using the ChamQ Universal SYBR qPCR Master (Vazyme Biotech Co., China) following the manufacturer’s instructions. The primers of qPCR were exquisitely selected to ensure only one peak per primer set, and the Cq values of all amplification curves were in the range from 15 to 30. Each sample was in triplicate and analyzed using Bio-Rad CFX Maestro (version 1.1) with normalized mode (ΔΔCq).

### Microscopy

An Olympus BX51 microscope (Tokyo) and a Retiga 2000R CCD camera (QImaging Corporation) were used to visualize cell morphology and green fluorescent protein. The images were acquired using QCapture Suite (QImaging Corporation).

## Data Availability

RNA-Seq data can be found in the supplemental material. Raw data have been deposited in the NCBI GEO repository under accession no. GSE307941.

## SUPPLEMENTAL MATERIAL

Table S1 msphere.00477-25-s0001.xlsx Differentially expressed genes in Ylrep1 cells.

Table S2 msphere.00477-25-s0002.xlsx Differentially expressed genes in Ylngs1 cells.

Supplemental Figures and Tables msphere.00477-25-s0003.pdf Fig. S1 and S2; Tables S3 to S6.

ASM does not own the copyrights to Supplemental Material that may be linked to, or accessed through, an article. The authors have granted ASM a non-exclusive, world-wide license to publish the Supplemental Material files. Please contact the corresponding author directly for reuse.
