# Supplementary material for: GlcNAc induces GlcNAc catabolic genes and inhibits filamentation via YlRep1-YlNgs1 signaling in the dimorphic yeast Yarrowia lipolytica
Source: mSphere. 2025 Oct 3;10(10):e00477-25. doi: 10.1128/msphere.00477-25 (PMC12570498; doi:10.1128/msphere.00477-25)
Supplement: Supplemental Figures and Tables — Fig. S1 and S2; Tables S3 to S6. [file msphere.00477-25-s0003.pdf]

Supplemental material

FIG S1. Cells of wild-type strains PO1a and W29 exhibited the same morphology in GlcNAc medium.

FIG S2. YlRep1 shares a large portion of target genes with YlNgs1.

TABLE S1. Differentially expressed genes in Yl*rep1*Δ and wild-type strains carrying pYL27-4NAG.

TABLE S2. Differentially expressed genes in Yl*ngs1*Δ and wild-type strains carrying pYL27-4NAG.

TABLE S3. Summary of the studies on the effect of GlcNAc on filamentation in *Y. lipolytica*.

TABLE S4. *Y. lipolytica* and *S. cerevisiae* strains used in this study.

TABLE S5. Plasmids used in this study.

TABLE S6. Oligonucleotides used in this study.

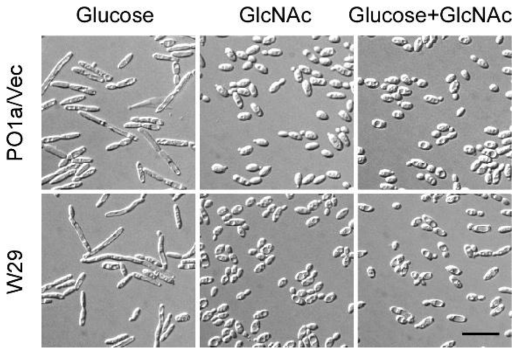

FIG S1. Cells of wild-type strains PO1a and W29 exhibited the same morphology in GlcNAc medium. Wild-type strain PO1a carrying empty vectors pINA445 (*CEN*, Yl*LEU2*) and pINA443 (*CEN*, Yl*URA3*) and strain W29 were grown in liquid YNB-Glucose, YNB-GlcNAc, or YNB-Glucose+GlcNAc medium for 16 h at 30°C. Bar, 20 μm.

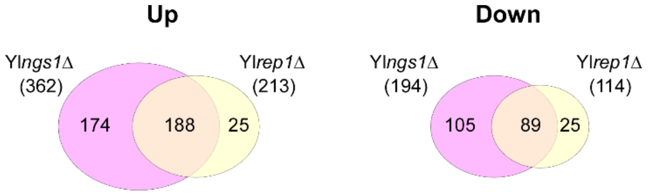

FIG S2. YlRep1 shares a large portion of target genes with YlNgs1. The Venn diagrams that represent the numbers of total, unique, and shared upregulated (Up) and downregulated (Down) genes between Yl*rep1*Δ and Yl*ngs1*Δ strains carrying pYL27-4NAG (P_{YlACT1}-*4NAG*) were shown.

TABLE S3. Summary of the studies on the effect of GlcNAc on filamentation in *Y. lipolytica.*

Strain	GlcNAc (w/v)	Buffer	pH	Other treatment	Temp.	Cell morphology	Filaments (%)	Source
**Studies suggesting that GlcNAc induces filamentation**								
CX-39-74C[a]	1%	50 mM citric acid-sodium citrate buffer	6.0	No	24°C	Hyphae (long filaments)	80-95%	Rodriguez and Dominguez (1984)
CX 39-74A	1%	50 mM citric acid-sodium citrate buffer	6.0	Cold treatment (4°C 15 min) and heat shock (30°C incubation)	30°C	Hyphae (long filaments)	~90%	Guevara-Olvera et al. (1993)
SA-1[a]	1%	50 mM citric acid-sodium citrate buffer	6.0	Nitrogen starvation and cold treatment (4°C, 2 h)	28°C	Hyphae (long filaments)	> 90%	Pérez-Campo and Domínguez (2001)
W29	1%	100 mM citrate buffer	7.0	No	27°C	A mixture of yeast form and filaments	NA	Braga et al. (2016)
**Studies suggesting that GlcNAc is not a good inducer of filamentation or GlcNAc inhibits filamentation**								
W29	1%	50 mM Phosphate buffer	7.0	No	28°C	Predominantly yeast form	8% (very short cells)	Ruiz-Herrera and Sentandreu (2002)
		100 mM Phosphate buffer	7.0	No	28°C	Predominantly yeast form	15% (very short cells)	
		50 mM Tris-HCl buffer	7.0	No	28°C	All yeast form	0%	
		100 mM Tris-HCl buffer	7.0	No	28°C	Predominantly yeast form	7% (very short cells)	
		50 mM citrate buffer	7.0	No	28°C	A mixture of yeast form and hyphae	54%	
		100 mM citrate buffer	7.0	No	28°C	Hyphae	91%	
PO1a	2%	No buffer	NA	No	30°C	Yeast form	0%	Flores and Gancedo (2015)
PO1a	1%	No buffer	~5.4	No	30°C	All yeast form	0%	This study
		Na_2HPO_4-citric acid buffer (63 µM citric acid)	7.0	No		Predominantly elongated cell and a few filaments	7% (> 20 µm) 65% (> 10 µm)	
		Na_2HPO_4-citric acid buffer (1.14 mM citric acid)	7.5	No		Predominantly long filaments and a few yeast form	82% (> 20 µm)	

Note: All the listed media contain 0.67% Yeast Nitrogen Base (YNB). a: Glucose did not induce filamentation in strains CX-39-74C and SA-1. NA: Not available.

References:

1. Braga A, Mesquita DP, Amaral AL, Ferreira EC, Belo I. (2016) Quantitative image analysis as a tool for *Yarrowia lipolytica*

dimorphic growth evaluation in different culture media. *J Biotechnol* 217:22-30.

2. Flores CL, Gancedo C. (2015) The gene *YALI0E20207g* from *Yarrowia lipolytica* encodes an N-acetylglucosamine kinase implicated in the regulated expression of the genes from the N-acetylglucosamine assimilatory pathway. *PLoS One* 10:e0122135.

3. Guevara-Olvera L, Calvo-Me´ndez C, Ruiz-Herrera J (1993) The role of polyamine metabolism in dimorphism of *Yarrowia lipolytica*. *J Gen Microbiol* 193:485-493.

4. Pérez-Campo FM, Domínguez A. (2001) Factors affecting the morphogenetic switch in *Yarrowia lipolytica*. *Curr Microbiol* 43:429-33.

5. Rodriguez C, Dominguez A. (1984) The growth characteristics of *Saccharomycopsis lipolytica*: morphology and induction of mycelial formation. *Can J Microbiol* 30:605–612.

6. Ruiz-Herrera J, Sentandreu R. (2002) Different effectors of dimorphism in *Yarrowia lipolytica*. *Arch Microbiol* 178:477-83.

TABLE S4. *Y. lipolytica* and *S. cerevisiae* strains used in this study.

Strain	Genotype	Source
Y. lipolytica strains		
PO1a	*MATA leu2-270 ura3-302*	1
YLX556	As PO1a except Yl*rep1Δ::loxR/P*	This study
YLX557	As PO1a except Yl*ngs1Δ::loxR/P*	This study
YLX558	As PO1a except Yl*ndt80Δ::loxR/P*	This study
YLX559	As PO1a except Yl*ngt1Δ::loxR/P*	This study
YLX560	As PO1a except Yl*nag5Δ::loxR/P*	This study
YLX561	As PO1a except Yl*dac1Δ::loxR/P*	This study
YLX562	As PO1a except Yl*nag1Δ::loxR/P*	This study
YLX563	As PO1a except Yl*rep1Δ::loxR/P* Yl*nag5Δ::loxR/P*	This study
YLX564	As PO1a except Yl*nag5Δ::loxR/P* Yl*dac1Δ::loxR/P* Yl*nag1Δ::loxR/P*	This study
S. cerevisiae strains		
pJ69-4A	*MATa his3-Δ200 leu2-3,112 trp1-901 ura3-52 gal4Δ gal80Δ LYS2::GAL1-HIS3 GAL2-ADE2 met2::GAL7-lacZ*	2
pJ69-4α	*MATα his3-Δ200 leu2-3,112 trp1-901 ura3-52 gal4Δ gal80Δ LYS2::GAL1-HIS3 GAL2-ADE2 met2::GAL7-lacZ*	2

References

1. Barth G and Gaillardin C (1996) The dimorphic fungus *Yarrowia lipolytica*. p.313-368. In K. Wolf (ed). Non-conventional yeasts in biotechnology. Springer, Heidelberg, Germany

2. James P, Halladay J, Craig EA (1996) Genomic libraries and a host strain designed for highly efficient two-hybrid selection in yeast. *Genetics* 144:1425-1436.

TABLE S5. Plasmids used in this study.

Plasmid	Description	Source
pINA445	pBR322 carrying *ARS68 (CEN/ARS)* and Yl*LEU2*	1
pINA445-YlREP1	Yl*REP1* carrying 2000-bp promoter and 400-bp 3’-UTR	This study
pINA445-YlNGS1	Yl*NGS1* carrying 1400-bp promoter and 400-bp 3’-UTR	This study
pINA445-YlNDT80	Yl*NDT80* carrying 500-bp promoter and 500-bp 3’-UTR	This study
pINA445-YlNGT1	Yl*NGT1* carrying 2000-bp promoter and 500-bp 3’-UTR	This study
pINA445-YlNAG5	Yl*NAG5* carrying 841-bp promoter and 500-bp 3’-UTR	This study
pINA445-YlDAC1	Yl*DAC1* carrying 2000-bp promoter and 517-bp 3’-UTR	This study
pINA445-YlNAG1	Yl*NAG1* carrying 2000-bp promoter and 500-bp 3’-UTR	This study
pINA445-lacZ	pINA445 carying lacZ reporter	2
pINA445-P_{YlNGT1}-lacZ	pINA445-lacZ carrying 3428-bp Yl*NGT1* promoter	This study
pINA445-P_{YlNAG5}-lacZ	pINA445-lacZ carrying 1000-bp Yl*NAG5* promoter	This study
pINA445-P_{YlDAC1}-lacZ	pINA445-lacZ carrying 1000-bp Yl*DAC1* promoter	This study
pINA445-P_{YlNAG1}-lacZ	pINA445-lacZ carrying 1000-bp Yl*NAG1* promoter	This study
pYL14	pINA445 carrying *EGFP*-T_{YlURA3}	2
pYL14-YALI0C23452$^{\Delta C}$	*YALI0C23452*$^{1\text{-}794}$*-GFP* (a.a. 1-794) carrying 2674-bp *YALI0C23452* promoter	This study
pYL14-YALI0E18722$^{\Delta C}$	*YALI0E18722*$^{1\text{-}608}$*-GFP* (a.a. 1-608) carrying 1775-bp *YALI0E18722* promoter	This study
pYL14-YALI0A00176$^{\Delta C}$	*YALI0A00176*$^{1\text{-}825}$*-GFP* (a.a. 1-829) carrying 2000-bp *YALI0A00176* promoter	This study
pYL13	pINA445 carrying 406-bp Yl*TEF1* promoter	2
pYL13-MHY1	P_{YlTEF1}-*MHY1* plus 1185-bp 3’-UTR	3
pYL13-YlWOR4	P_{YlTEF1}-Yl*WOR4* plus 300-bp 3’-UTR	This study
pYL13-YlCBF1	P_{YlTEF1}- Yl*CBF1* plus 300-bp 3’-UTR	This study
pYL13-YlAAF2	P_{YlTEF1}- Yl*AAF2* plus 300-bp 3’-UTR	This study
pYL8	pBlueScript KS(+) carrying *loxR*-Yl*URA3-loxP*	2
pYL8-YlREP1	P_{YlREP1}-*loxR*-Yl*URA3-loxP*-T_{YlREP1} in pYL8	This study
pYL8-YlNGS1	P_{YlNGS1}-*loxR*-Yl*URA3-loxP*-T_{YlNGS1} in pYL8	This study
pYL8-YlNDT80	$P_{YlNDT80}$-*loxR*-Yl*URA3-loxP*-$T_{YlNDT80}$ in pYL8	This study
pYL8-YlNGT1	P_{YlNGT1}-*loxR*-Yl*URA3-loxP*-T_{YlNGT1} in pYL8	This study
pYL8-YlNAG5	P_{YlNAG5}-*loxR*-Yl*URA3-loxP*-T_{YlNAG5} in pYL8	This study
pYL8-YlDAC1	P_{YlDAC1}-*loxR*-Yl*URA3-loxP*-T_{YlDAC1} in pYL8	This study
pYL8-YlNAG1	P_{YlNAG1}-*loxR*-Yl*URA3-loxP*-T_{YlNAG1} in pYL8	This study
pRRQ2	*ARS68 (CEN/ARS)*, Yl*LEU2*, *hp4d-CRE*	4
pYL21	406-bp Yl*TEF1* promoter, *ARS68 (CEN/ARS)*,	Lab collection

	Yl*URA3*	
pYL21-lexA	P_{YlTEF1}-*lexA* containing 254-bp Yl*TEF1* promoter and *lexA* (a.a. 1-87)	This study
pYL21-lexA-YlREP1	P_{YlTEF1}- *lexA-YlREP1* containing 254-bp Yl*TEF1* promoter and *lexA* (a.a. 1-87)	This study
pYL21-lexA-YlNGS1	P_{YlTEF1}- *lexA-YlNGS1* containing 254-bp Yl*TEF1* promoter and *lexA* (a.a. 1-87)	This study
pINA445-lexAop-P_{YlLEU2}-lacZ	*lexAop4-*P_{YlLEU2}*-lacZ* in pINA445	2
pYL25	pYL21 carrying the 947-bp Yl*ACT1* promoter instead of Yl*TEF1* promoter	This study
pYL25-4NAG	P_{YlACT1}-Yl*NGT1,* P_{YlACT1}-Yl*NAG5,* P_{YlACT1}-Yl*DAC1,* P_{YlACT1}-Yl*NAG1*	This study
pYL27	pINA445 carrying the 947-bp Yl*ACT1* promoter	This study
pYL27-4NAG	P_{YlACT1}-Yl*NGT1,* P_{YlACT1}-Yl*NAG5,* P_{YlACT1}-Yl*DAC1,* P_{YlACT1}-Yl*NAG1*	This study
pGAD-C1	2μ, *LEU2, GAL4-AD*	5
pGAD-YlNGS1	Yl*NGS1* in pGAD-C1	This study
pGAD-YlREP1	Yl*REP1* in pGAD-C1	This study
pGAD-YlREP1$^{1-200}$	Yl*REP1*$^{1-200}$ in pGAD-C1	This study
pGAD-YlREP1$^{1-376}$	Yl*REP1*$^{1-376}$ in pGAD-C1	This study
pGAD-YlREP1$^{377-494}$	Yl*REP1*$^{377-494}$ in pGAD-C1	This study
pGBDU-C1	2μ, *URA3, GAL4-BD*	5
pGBDU -YlNGS1	Yl*NGS1* in pGBDU-C1	This study
pGBDU -YlREP1	Yl*REP1* in pGBDU-C1	This study
pGBDU -YlREP1$^{1-200}$	Yl*REP1*$^{1-200}$ in pGBDU-C1	This study
pGBDU -YlREP1$^{1-376}$	Yl*REP1*$^{1-376}$ in pGBDU-C1	This study
pGBDU -YlREP1$^{377-494}$	Yl*REP1*$^{377-494}$ in pGBDU-C1	This study

References

1. Nuttley WM, Brade AM, Gaillardin C, Eitzen GA, Glover JR, Aitchison JD and Rachubinski RA (1993) Rapid identification and characterization of peroxisomal assembly mutants in *Yarrowia lipolytica*. *Yeast*. 9: 507–517.

2. Zhao X-F, Li M, Li Y-Q, Chen X-D and Gao X-D (2013) The TEA/ATTS transcription factor YlTec1p represses the yeast-to-hypha transition in the dimorphic yeast *Yarrowia lipolytica*. *FEMS Yeast Res*. 13: 50-61.

3. Wu H, Shu T, Mao Y-S and Gao X-D (2020) Characterization of the promoter, downstream target genes and recognition DNA sequenced of Mhy1, a key filamentation-promoting transcription factor in the dimorphic yeast *Yarrowia lipolytica*. *Curr. Genet*. 66: 245-261.

4. Richard M, Quijano RR, Bezzate S, Bordon-Pallier F and Gaillardin C (2001) Tagging morphogenetic genes by insertional mutagenesis in the yeast *Yarrowia lipolytica*. *J. Bacteriol*. 183: 3098-3107.

5. James P, Halladay J and Craig EA (1996) Genomic libraries and a host strain designed for highly efficient two-hybrid selection in yeast. *Genetics* 114: 1425-1436.

TABLE S6. Oligonucleotides used in this study.

Name	Sequence (5’→3’)	Use
YlREP1-PF	cgctctagaactagtggatccACCTGTCAGCTGGCGGTTT	Amplification of P_{YlREP1}
YlREP1-PR	ttcctgcagcccgggggatccTTGTGGTGGCAGTTGGTGG	Amplification of P_{YlREP1}
YlREP1-TF	gctatacgaagttataagcttGGCTGGAGATGTACAAGACAA GAA	Amplification of T_{YlREP1}
YlREP1-TR	gtcgacggtatcgataagcttTGAACAATGCTCTGAACTACT GTATGTG	Amplification of T_{YlREP1}
YlREP1-5CK	AAGCGAAGGCAGGCGAGTATCAG	Checking Yl*rep1*Δ deletion
YlREP1-3CK	AAGACAAAGAAGGGCTGCTCAAG	Checking Yl*rep1*Δ deletion
YlREP1-F	cagcttatcatcgataagcttGACATAGTTGTTACCACCCTCA TTTT	Amplification of Yl*REP1*
YlREP1-R	taaactaccgcattaaagcttGCCAAGAGCGCTGCTCCG	Amplification of Yl*REP1*
YlNGS1-PF	ggcggccgctctagaactagtGCGTCTTTTCCATCTCCA	Amplification of P_{YlNGS1}
YlNGS1-PR	cagcccgggggatccactagtTTGTGATGGGGTGGGAAT	Amplification of P_{YlNGS1}
YlNGS1-TF	gctatacgaagttataagcttCCCGCATCTATTGTATGT	Amplification of T_{YlNGS1}
YlNGS1-TR	gtcgacggtatcgataagcttAGCGCTCAAAGTCGTACA	Amplification of T_{YlNGS1}
YlNGS1-5CK	ACGTTGGCGTAGGTGGAAGGCAG	Checking Yl*ngs1*Δ deletion
YlNGS1-3CK	TGTATGTGTGGTGAATGCGGAGG	Checking Yl*ngs1*Δ deletion
YlNGS1-F	gggcgccatctccttgcatgcTCATGGAGGCTCCTCCCTG	Amplification of Yl*NGS1*
YlNGS1-R	gccgcaaggaatggtgcatgcAAGTAGCTACCAGTATTGTAT TAGCCAAA	Amplification of Yl*NGS1*
YlNDT80-PF	ggcggccgctctagaactagtATGAAAGTCAGGATAGCCTTA CCC	Amplification of $P_{YlNDT80}$
YlNDT80-PR	cagcccgggggatccactagtTATCTGTTGTGTAGGGATGTT TTTTTC	Amplification of $P_{YlNDT80}$
YlNDT80-TF	gctatacgaagttataagcttGTTGGTGGCCCATGTATCGC	Amplification of $T_{YlNDT80}$
YlNDT80-TR	gtcgacggtatcgataagcttGGTTAGGTTGACCTTTAACCA GTAAA	Amplification of $T_{YlNDT80}$
YlNDT80-5CK	CGGTAAGAGCAACTGTGCTCTTG	Checking Yl*ndt80*Δ deletion
YlNDT80-3CK	CACCAAGTCTGGTGATGCCTCCG	Checking Yl*ndt80*Δ deletion
YlNDT80-F	cagcttatcatcgataagcttCTGCAGAGAGACAAACAGCTC AA	Amplification of Yl*NDT80*
YlNDT80-R	taaactaccgcattaaagcttTTCCACTCAAACTGTGGATCGA	Amplification of Yl*NDT80*
YlNGT1-PF	ggcggccgctctagaactagtTGATCAGCTATCTACGAGGGC TATT	Amplification of P_{YlNGT1}
YlNGT1-PR	cagcccgggggatccactagtTTTGACTTTAATTTTAGACTT GTTAGGTTC	Amplification of P_{YlNGT1}
YlNGT1-TF	gctatacgaagttataagcttGGAGCATTAGGACTTCTGGGTG	Amplification of T_{YlNGT1}
YlNGT1-TR	gtcgacggtatcgataagcttTGTCCCAACCCGATTGAGAA	Amplification of T_{YlNGT1}
YlNGT1-5CK	TATCGAGAGTGCGAGTAAGCCAG	Checking Yl*ngt1*Δ deletion
YlNGT1-3CK	CTCCTCGTGGCTACATGGTCATG	Checking Yl*ngt1*Δ deletion
YlNGT1-F	cagcttatcatcgataagcttATGGTGGTTTTTACCGGCCC	Amplification of Yl*NGT1*

YlNGT1-R	taaactaccgcattaaagcttCAAATGCAATTGTTGCCGTG	Amplification of Yl*NGT1*
YlNAG5-PF	tcccccgggctgcaggaattcTGATTTGATAATGCAAGGGCTTG	Amplification of P_{YlNAG5}
YlNAG5-PR	gttatccgaagcgatgaattcATATAAATATCCTCTGCGCTCGATG	Amplification of P_{YlNAG5}
YlNAG5-TF	cgaagttataagcttatcgatATAGCATTCGACCAAAATGACGA	Amplification of T_{YlNAG5}
YlNAG5-TR	ctcgaggtcgacggtatcgatGGTAGAGGAGATTGAAGCCAGGG	Amplification of T_{YlNAG5}
YlNAG5-5CK	TCCATAGCCCGGTGGCTGCCTTG	Checking Yl*nag5*Δ deletion
YlNAG5-3CK	TTCAGAGACGCCACTTGTAACAG	Checking Yl*nag5*Δ deletion
YlNAG5-F	cagcttatcatcgataagcttGCCCGCATCTATTGTATGTATTTG	Amplification of Yl*NAG5*
YlNAG5-R	taaactaccgcattaaagcttGGATACTATGCATGCAAAGATTGAA	Amplification of Yl*NAG5*
YlDAC1-PF	tcccccgggctgcaggaattcGGTGTCTTTGGAGGCTGCC	Amplification of P_{YlDAC1}
YlDAC1-PR	gttatccgaagcgatgaattcGGCGAGAGGTTTCGAGAGCC	Amplification of P_{YlDAC1}
YlDAC1-TF	cgaagttataagcttatcgatGGGAGGACGACACACGACG	Amplification of T_{YlDAC1}
YlDAC1-TR	ctcgaggtcgacggtatcgatCCCCTCCTCAGACCTGCCC	Amplification of T_{YlDAC1}
YlDAC1-5CK	TCGTGATCGTCTCCAAAGGC	Checking Yl*dac1*Δ deletion
YlDAC1-3CK	ACCCAACCCGAGTATTGACG	Checking Yl*dac1*Δ deletion
YlDAC1-F	cagcttatcatcgataagcttCAATGGTCTTTCCCAGATGTTGA	Amplification of Yl*DAC1*
YlDAC1-R	taaactaccgcattaaagcttCCCGGTTTGCGCCCATTA	Amplification of Yl*DAC1*
YlNAG1-PF	tcccccgggctgcaggaattcAGACATGTTGACCTCGTACACTAAATT	Amplification of P_{YlNAG1}
YlNAG1-PR	gttatccgaagcgatgaattcTGTGCTGGTGGGTATTGAGCT	Amplification of P_{YlNAG1}
YlNAG1-TF	cgaagttataagcttatcgatACGATGCAAAGTCATTGTCCATC	Amplification of T_{YlNAG1}
YlNAG1-TR	ctcgaggtcgacggtatcgatTTTCGAAGCTCAAAGTCGTGC	Amplification of T_{YlNAG1}
YlNAG1-5CK	GAGGAATAGTATCGCGACTCGT	Checking Yl*nag1*Δ deletion
YlNAG1-3CK	GGCCGTTTTTCGAAGCTCAA	Checking Yl*nag1*Δ deletion
YlNAG1-F	cagcttatcatcgataagcttTCTCAGAGGGTATCTAGGGTGGC	Amplification of Yl*NAG1*
YlNAG1-R	taaactaccgcattaaagcttGATCCTTGGATGTTGTCAGAAGG	Amplification of Yl*NAG1*
P_{YlNGT1}-F	agaactagtggatccaagcttTGGCCCTACTTGCTGCTGG	Amplification of P_{YlNGT1} plus ATG
P_{YlNGT1}-R	cagggtaccgtcgacaagcttCATTTTGACTTTAATTTTAGACTTGTTAGG	Amplification of P_{YlNGT1} plus ATG
P_{YlNAG5}-F	agaactagtggatccaagcttTGATTTGATAATGCAAGGGCTTG	Amplification of P_{YlNAG5} plus ATG
P_{YlNAG5}-R	cagggtaccgtcgacaagcttCATATATAAATATCCTCTGCGCTCG	Amplification of P_{YlNAG5} plus ATG

P_{YlDAC1}-F	agaactagtggatccaagcttGGTGTCTTTGGAGGCTGCC	Amplification of P_{YlDAC1} plus ATG
P_{YlDAC1}-R	cagggtaccgtcgacaagcttCATGGCGAGAGGTTTCGAGA	Amplification of P_{YlDAC1} plus ATG
P_{YlNAG1}-F	agaactagtggatccaagcttAGACATGTTGACCTCGTACACTAAAT	Amplification of P_{YlNAG1} plus ATG
P_{YlNAG1}-R	cagggtaccgtcgacaagcttCATTGTGCTGGTGGGTATTGAG	Amplification of P_{YlNAG1} plus ATG
YALI0C23452-GFPF	agatctagaactagtggatccGACAACCTAAATAGATAATTTGATGTGTATAGA	Amplification of YALI0C23452$^{1-794}$
YALI0C23452-GFPR	accgtcgacaagcttggatccCTCGGGAGTGTTACTTCCACC	Amplification of YALI0C23452$^{1-794}$
YALI0E18722-GFPF	agatctagaactagtggatccGTCCATACAGACAACCGATATCTTCG	Amplification of YALI0E18722$^{1-608}$
YALI0E18722-GFPR	accgtcgacaagcttggatccCTGGGCAACGGTGGTGC	Amplification of YALI0E18722$^{1-608}$
YALI0A00176-GFPF	agatctagaactagtggatccTGTTTCTGGATACTTCATCACTTCG	Amplification of YALI0A00176$^{1-825}$
YALI0A00176-GFPR	accgtcgacaagcttggatccGTCGGAAGGCACGGGC	Amplification of YALI0A00176$^{1-825}$
YlWOR4-OF	caggaattcgatatcaagcttATGTATATAGATATATATATATATCAATGGCTCACG	Amplification of Yl*WOR4* ORF
YlWOR4-OR	gtcgacggtatcgataagcttCATAGGGTCTAGATCAGGGACGTT	Amplification of Yl*WOR4* ORF
YlCBF1-OF	caggaattcgatatcaagcttATGGACCTCAAATCAATTGTGTTG	Amplification of Yl*CBF1* ORF
YlCBF1-OR	gtcgacggtatcgataagcttCACTGTACAGGTGCCAGTACATACC	Amplification of Yl*CBF1* ORF
LexA-HF	caggaattcgatatcaagcttATGAAAGCGTTAACGGCCAG	Amplification of *lexA* (a.a. 1-87)
LexA-HR	gtcgacggtatcgataagcttTGGTTCACCGGCAGCCAC	Amplification of *lexA* (a.a. 1-87)
LexA-YlREP1-F	GTAGGTCGTGTGGCTGCCGGTGAACCAATGACATCACCACATTTTGATGACTCCGG	Overlapping PCR fusion of *lexA* (a.a. 1-87) and Yl*REP1*
YlREP1-LexA-R	CCGGAGTCATCAAAATGTGGTGATGTCATTGGTTCACCGGCAGCCACACGACCTAC	Overlapping PCR fusion of *lexA* (a.a. 1-87) and Yl*REP1*
YlREP1-HR	gtcgacggtatcgataagcttGGCCGGTCTTTTGGCCGG	Amplification of LexA-Yl*REP1* ORF
LexA-YlNGS1-F	GTAGGTCGTGTGGCTGCCGGTGAACCAATGGAGGAATTATCGGAGGCGGAACG	Overlapping PCR fusion of *lexA* (a.a. 1-87) and Yl*NGS1*
YlNGS1-LexA-R	CGTTCCGCCTCCGATAATTCCTCCATTGGTTCACCGGCAGCCACACGACCTAC	Overlapping PCR fusion of *lexA* (a.a. 1-87) and Yl*NGS1*
YlNGS1-HR	gtcgacggtatcgataagcttGCGCCATCGGGGTTGGTG	Amplification of LexA-

		Yl*NGS1* ORF
P_{YlACT1}-F	ACAATGTGCTGGCATTTACTTGTGCTGGC	Amplification of Yl*ACT1* promoter
P_{YlACT1}-R	TAATTTGTGTGGTTGTTGGTAGCAAAGAG	Amplification of Yl*ACT1* promoter
YlNGS1-ADF	atcgaattccccgggggatccATGGAGGAATTATCGGAGGCG	Amplification of Yl*NGS1* ORF for yeat two-hybrid
YlNGS1-ADR	caggtcgacatcgatggatccCTACCGGCTGAACTTCTCTTCC	Amplification of Yl*NGS1* ORF for yeat two-hybrid
YlNGS1-BDF	ccggaattccccgggggatccATGGAGGAATTATCGGAGGCG	Amplification of Yl*NGS1* ORF for yeat two-hybrid
YlNGS1-BDR	caggtcgacatcgatggatccCTACCGGCTGAACTTCTCTTCC	Amplification of Yl*NGS1* ORF for yeat two-hybrid
YlREP1-ADF	atcgaattccccgggggatccATGACATCACCACATTTTGATGACT	Amplification of Yl*REP1* ORF for yeat two-hybrid
YlREP1-ADR	caggtcgacatcgatggatccCTACTCAATCTTGGAGAAATACTTTCG	Amplification of Yl*REP1* ORF for yeat two-hybrid
YlREP1-BDF	ccggaattccccgggggatccATGACATCACCACATTTTGATGACT	Amplification of Yl*REP1* ORF for yeat two-hybrid
YlREP1-BDR	caggtcgacatcgatggatccCTACTCAATCTTGGAGAAATACTTTCG	Amplification of Yl*REP1* ORF for yeat two-hybrid
YlREP1-N200-ADR	caggtcgacatcgatggatccCATTTTGGCCACGTCGCC	Amplification of Yl$REP1^{1-200}$ for yeat two-hybrid
YlREP1-N200-BDR	caggtcgacatcgatggatccCATTTTGGCCACGTCGCC	Amplification of Yl$REP1^{1-200}$ for yeat two-hybrid
YlREP1-N376-ADR	caggtcgacatcgatggatccTCGGTTGAGGTAGAACCGG	Amplification of Yl$REP1^{1-376}$ for yeat two-hybrid
YlREP1-N376-BDR	caggtcgacatcgatggatccTCGGTTGAGGTAGAACCGG	Amplification of Yl$REP1^{1-376}$ for yeat two-hybrid
YlREP1-C377-ADF	atcgaattccccgggggatccAACTGCATCATTTTGAACGATGG	Amplification of Yl$REP1^{377-494}$ for yeat two-hybrid
YlREP1-C377-BDF	ccggaattccccgggggatccAACTGCATCATTTTGAACGATGG	Amplification of Yl$REP1^{377-494}$ for yeat two-hybrid
YlNGT1-qF	TGACTGGTCCGACTCTCGAT	qPCR for Yl*NGT1*
YlNGT1-qR	AGCAGAGTTGGAAAGGGCTC	qPCR for Yl*NGT1*
YlNAG5-qF	TTCGAGACCCACTTTGCCTC	qPCR for Yl*NAG5*
YlNAG5-qR	TTGATGCCTGTGCCGAGAAT	qPCR for Yl*NAG5*
YlDAC1-qF	TTGGTATTCACGTCGAGGGC	qPCR for Yl*DAC1*
YlDAC1-qR	TGATGACACGGACGTTCTGG	qPCR for Yl*DAC1*
YlNAG1-qF	CGACAGGTGTTTCTGGACGA	qPCR for Yl*NAG1*
YlNAG1-qR	GGCAAAGCCAAAGGCAAGAA	qPCR for Yl*NAG1*
GAPDH-qF	CCGAGTCCCTACCGTTGATG	qPCR for *GAPDH*

GAPDH-qR	TCATGGTGGCCTTGATGTCC	qPCR for *GAPDH*
YlWOR4-qF	CTGCCGCTCAACAAAAACCA	qPCR for Yl*WOR4*
YlWOR4-qR	TGCCATAGGCCTTGGAACAG	qPCR for Yl*WOR4*
MHY1-qF	TCGAGTATGCACACGACGAG	qPCR for *MHY1*
MHY1-qR	TCGAGAGGCGAGTAAGTGGA	qPCR for *MHY1*
YlCBF1-qF	ACCAGACACTGAACACAGCC	qPCR for Yl*CBF1*
YlCBF1-qR	TAGGGCAGCCATGGACTTTG	qPCR for Yl*CBF1*
YlAAF2-qF	GCTACTCTGCGCCACAAAAC	qPCR for Yl*AAF2*
YlAAF2-qR	GGTGAACTTGGACCGGTAGG	qPCR for Yl*AAF2*
YALI0F07535-qF	ACTCCCTCTCTGACGCTGAT	qPCR for *YALI0F07535*
YALI0F07535-qR	CAAGGCCGATGGCAATAACG	qPCR for *YALI0F07535*
YALI0E18722-qF	AGATCTGCTGCGGTTTGTCA	qPCR for *YALI0E18722*
YALI0E18722-qR	AAGCAACAGCATCGGAGGAA	qPCR for *YALI0E18722*
YALI0C23452-qF	TGCGACACTACCACCACTTC	qPCR for *YALI0C23452*
YALI0C23452-qR	GAACTAGTGGGCACAGGCTT	qPCR for *YALI0C23452*
YALI0A00176-qF	ACCCCTACCCCCACTGATAC	qPCR for *YALI0A00176*
YALI0A00176-qR	GAGTGGTTCCGAGCTCAACA	qPCR for *YALI0A00176*
YALI0F21857-qF	AACGTGCCCAACTGTACCAA	qPCR for *YALI0F21857*
YALI0F21857-qR	GGAGAGACGGCAGTGTTGAA	qPCR for *YALI0F21857*
YALI0E31108-qF	ACGGTAAGCCCGTTGAGTTT	qPCR for *YALI0E31108*
YALI0E31108-qR	CGAATCGGGGAACGTAGAGG	qPCR for *YALI0E31108*
YALI0E20823-qF	CGTCTGCCTACGCTACTCTG	qPCR for *YALI0E20823*
YALI0E20823-qR	GATGACGGCCGATCCATCTT	qPCR for *YALI0E20823*

Note: Gene sequences are written in capital letters. Restriction sites used for cloning are underlined.
